# Novel Structural Parameters of Ig–Ag Complexes Yield a Quantitative Description of Interaction Specificity and Binding Affinity

**DOI:** 10.3389/fimmu.2017.00034

**Published:** 2017-02-09

**Authors:** Simon Marillet, Marie-Paule Lefranc, Pierre Boudinot, Frédéric Cazals

**Affiliations:** ^1^VIM, INRA and Université Paris-Saclay, Jouy-en-josas, France; ^2^Université Côte d’Azur and Inria, Sophia Antipolis, France; ^3^IMGT, IGH, CNRS, Montpellier, France

**Keywords:** antibody–antigen complex, molecular recognition, binding affinity prediction, complementarity determining region 3, interaction specificity

## Abstract

Antibody–antigen complexes challenge our understanding, as analyses to date failed to unveil the key determinants of binding affinity and interaction specificity. We partially fill this gap based on novel quantitative analyses using two standardized databases, the IMGT/3Dstructure-DB and the structure affinity benchmark. First, we introduce a statistical analysis of interfaces which enables the classification of ligand types (protein, peptide, and chemical; cross-validated classification error of 9.6%) and yield binding affinity predictions of unprecedented accuracy (median absolute error of 0.878 kcal/mol). Second, we exploit the contributions made by CDRs in terms of position at the interface and atomic packing properties to show that in general, VH CDR3 and VL CDR3 make dominant contributions to the binding affinity, a fact also shown to be consistent with the enthalpy–entropy compensation associated with preconfiguration of CDR3. Our work suggests that the affinity prediction problem could be partially solved from databases of high resolution crystal structures of complexes with known affinity.

## 1 Introduction

### 1.1 Immunoglobulins and the Immune Response

Adaptive immunity is based on antigen (Ag)-specific lymphocyte responses. Upon specific recognition of an antigenic epitope by a given receptor unique to a lymphocyte, this cell gets activated and proliferates, leading to a clonal expansion. B lymphocytes thus recognize antigens through membrane-bound immunoglobulins (Ig) expressed at their surface. Seric Igs can opsonize bacteria and facilitate their uptake by phagocytes or neutralize viruses thus preventing recognition by their receptor or fusion with the target cell. Immunoglobulins fundamentally consist of two identical heavy (H) chains and two identical light (L) chains, each H chain being bound to an L chain. The antigen-binding site is located at the top of the paired VH and VL, and generally overlaps the two V domains. It mainly consists of three flexible loops on each V domain, called complementarity determining regions (CDR1–3). The diversity of antibodies is concentrated in the CDRs.

From the structural standpoint, the functional relevance of an Ig depends on its binding affinity for the targeted antigen and the specificity of the interactions, which provides the basis of immune memory and vaccination. The affinity sets the strength of the interaction. For the membrane-bound Ig, it determines if enough aggregation of surface Igs and Ig co-receptors occurs, so that a sufficient signal can be sent to the cell to induce activation and proliferation (1). For secreted Ig, once bound to the target, pathogens or host infected/tumoral cells, the affinity sets the efficiency of Ig-mediated pathogen opsonization and/or neutralization, or Ig effector properties (antibody-dependent cell-cytotoxicity or ADCC, complement-dependent cytotoxicity or CDC) (2).

### 1.2 Ig–Ag Complexes and Underlying Genetic Mechanisms

The prominent role played in Ag binding by CDRs has prompted the analysis of CDR-specific statistics. Using a handful of crystallographic structures, canonical conformations, i.e., commonly occurring backbone CDR conformations were first reported (3) and subsequently updated (4, 5), using 300 non-redundant Ig structures in the latest work (6). Moving from individual CDR to all CDRs, correlations between canonical conformations were further studied (7), highlighting the fact that some combinations are multi-specific, while others are specific of an antigen type. The VH CDR3 is the most variable and was therefore the focus of several studies (8–10) which defined and updated sequence-based rules to predict its conformations. More recently, these studies have been refined, based on a larger number of structures (of the order of hundreds instead of tens). For VL CDR3, new canonical conformations were proposed (11), and for VH CDR3, previous rules were updated and complemented (12, 13). Distinguishing lambda versus kappa chains, it has been shown that canonical conformations from the former are more diverse than those from the latter in the human and the mouse (14). However, the relevance of canonical conformations for the prediction of the 3D structures of CDRs was questioned (15), since general loop prediction methods matched (or even outperformed) the prediction performances of methods exploiting specific rules associated with canonical conformations of CDRs. In parallel, two related works (16, 17) studied the differential CDR lengths and Specificity-Determining Residues Usage (SDRU, proportion of Ig amino acids at a given CDR position which contact the antigen) between ligand types. However, these analyses do not allow antigen type predictions. To assess the role of individual CDRs, it has also been established that except in the case of bacterial carbohydrates, diversity in VH CDR3 alone can result in primary responses specific to the antigen (18). Structural and genetic aspects of individual CDRs in natural and artificial antibody repertoires are reviewed in Ref. (19).

### 1.3 Ig–Ag Complexes and Thermodynamics

The analysis of Ig–Ag complexes can also be posed from the thermodynamics standpoint. Specifically, the binding affinity is a thermodynamic quantity describing the chemical equilibrium associated with the two partners (Ig and Ag in our case) and the complex [Ig–Ag, denoted IG/Ag in the IMGT nomenclature (20)]. It is generally measured by the dissociation constant *K_d_* (=[Ig]⋅[Ag]/[Ig–Ag]) of this equilibrium. Equivalently, it is expressed by the corresponding dissociation free energy

(1) $$\Delta G=-\mathrm{RT}\ln K_{d}∕c^{\circ }=\Delta H-T\Delta S,$$

in the *c*^∘^ = 1*M* standard state, with *T* the temperature and *R* the gas constant. Thus, by nature, the affinity has an enthalpic component (Δ*H*) qualifying the interaction energy, but also an entropic component (*T* Δ*S*) qualifying the loss of dynamical properties upon complex formation (intuitively, the formation of the Ig–Ag complex indeed restricts the degrees of freedom of both partners). These two competing interests illustrate the enthalpy–entropy compensation phenomenon (21, 22), which stipulates that a favorable enthalpic change upon association is accompanied by an entropic penalty. Predicting binding affinities from structural data requires to quantify this compensation and is therefore a notoriously challenging problem, for protein complexes in general (23, 24), and for Ig–Ag complexes in particular (25).

To model the enthalpic component, various parameters have been proposed. Most of these parameters, which describe the morphology of the interface (size, shape, and packing properties) and its biochemistry (salt bridges, solvation, and hydrogen bonds), were estimated from crystal structures of complexes (26–29). More recently, it has also been shown that non-interacting atoms play an important role, intuitively related to solvent interactions (30). Such approaches have been applied to Ig–Ag complexes (31), stressing in particular the role of interfacial solvent (32), biochemical properties of Igs as a function of epitopes (33), or the correlation between interface curvature and ligand size (34).

To model the entropic component, the conformational and vibrational properties of the partners must be captured. It has indeed been shown that the preconfiguration of the binding site may yield a decreased entropic loss, hence an enhanced binding affinity (35–38). It has also been shown that a preconfiguration of the variable domains can be induced by the constant domain 1 (CH1) of the heavy chain (39, 40), suggesting that the isotype switching commonly occurring during B cell differentiation may affect the affinity through changes in the dynamic properties of the Ig. Parallel to binding affinity, the notion of functional affinity or avidity which takes into account the (possibly negative) cooperativity between monomers of an antibody is highly relevant *in vivo*. In that context, constant regions have been shown to influence the avidity (41–44). Likewise, an intact ball-and-socket joint between VH and CH1 domains has been shown to affect antibody neutralizing activity (45).

### 1.4 Contributions

The difficulty of understanding molecular recognition between proteins in general and antibody–antigens in particular is well known (31). In this work, we present novel quantitative analyses for interfaces of Ig–Ag complexes. Using the annotated IMGT/3Dstructure-DB (20), the interface between the Ig chains and the Ag is determined using a Voronoi-based model for each complex and decomposed into contributions from CDR, framework (FR), and atoms outside the V region. This interface allows dissecting the interface into contributions made by CDRs, in terms of position of their atoms at the interface and packing properties of these atoms. Using these parameters, we show how to unambiguously distinguish ligand types and predict binding affinity with unprecedented accuracy. We also develop quantitative models for the contribution of VH CDR3 to binding affinity and interaction specificity, bridging the gap between various observations (canonical backbone conformations, mutagenesis data, and affinity measurements), and explaining the emergence of function from a combination of structural and dynamical properties.

## 2 Materials and Methods

### 2.1 Voronoi Interface Models

Given a macromolecular complex, an interface model is a structural model of the atoms accounting for the interactions, ideally encompassing its enthalpic (i.e., interaction energy) and entropic (i.e., dynamic) dimensions. In the sequel, we model complexes and their interfaces using solvent-accessible models (46) and the associated Voronoi-based interface model [Figure S3 in Supplementary Material; (47, 48)].

#### 2.1.1 Solvent-Accessible Models and Voronoi Interfaces

The *solvent-accessible model* (SAM) of a set of atoms is a model where each atom is represented by a ball whose radius is the van der Waals radius expanded by the radius *r_w_* = 1.4 Å of a water probe accounting for a continuous solvation layer (46, 49). A convenient construction to study SAM is the Voronoi (power) diagram defined by the atoms (49). In particular, the Voronoi diagram induces a partition of the molecular volume, obtained by computing for each atom its *Voronoi restriction*, namely, the intersection between its atomic ball and its Voronoi region. The volume of this restriction, also called atomic volume, is a direct measure of the atomic packing (49).

The *exposed surface* of a SAM consists of the boundary of the union of balls defining the SAM. This surface consists of spherical polygons, delimited by circle arcs (every such arc is located on the intersection circle of two atoms), themselves delimited by points (each such point is found at the intersection of three atoms). When two molecules assemble to form a complex, the *buried surface area* (BSA) is the portion of the exposed surface of both partners which gets buried (27). BSA has been shown to exhibit remarkable correlations with various biophysical quantities (50), and notably dissociation free energies for complexes involving moderate flexibility (29).

Consider the SAM of a complex whose partners are denoted A and B, and also involving interfacial water molecules W. Two atoms are in *contact* provided that their Voronoi restrictions are neighbors. Pairs of type (A, B) define the AB interface, namely, direct contacts between the partners. Focusing on water molecules W sandwiched between the partners, pairs (A, W) and (B, W) correspond to water mediated interactions. It can be shown that all atoms from the partners identified this way form a superset of atoms loosing solvent accessibility (51). The *binding patch* of a partner consists of its interface atoms. The atoms of the binding patch can be assigned an integer called its *shelling order*, which is a measure of the distance of this atom to the boundary of the patch it belongs to (28). This information generalizes the core–rim model (27) and has been shown to provide state-of-the-art correlations with solvent dynamics, conservation of amino acids (28), and dissociation free energies (29). All tools to compute the parameters just discussed are available within the Structural Bioinformatics Library at http://sbl.inria.fr > Applications > Space Filling Models.

#### 2.1.2 Application to Ig–Ag Complexes

For an Ig–Ag complex, we partition the set I of interface atoms just defined into the atoms I_Ig_ contributed by the Ig, and the atoms I_Ag_ contributed by the Ag, so that $I=I_{\text{Ig}}\cup I_{\text{Ag}}$. It follows that the number of interface atoms |I| is the sum of those contributed by the Ig and the Ag, respectively, namely, |I| = |I_Ig_| + |I_Ag_|. Similarly, we charge the Buried Surface Area (BSA) to the Ig and Ag, respectively, so that BSA = BSA_Ig_ + BSA_Ag_. These quantities yield the average BSA per interface atom on Ig and Ag side:

(2) $${\bar{\text{bsa}}}_{\text{Ig}}=\frac{\text{BS}{\text{A}}_{\text{Ig}}}{\left|I_{\text{Ig}}\right|},$$

(3) $${\bar{\text{bsa}}}_{\text{Ag}}=\frac{\text{BS}{\text{A}}_{\text{Ag}}}{\left|I_{\text{Ag}}\right|}.$$

The previous analysis can be generalized to accommodate the structure of Fabs, by decomposing the variable domains of each chain (VH and VL) into to three complementarity determining regions (CDRs) and four framework regions (FRs), resulting in 14 Voronoi interfaces. Practically, we focus on contacts made by the six CDRs, those made by framework regions being negligible (Table S5 in Supplementary Material). (Details of the method used at http://sbl.inria.fr/doc/Space_filling_model_interface-user-manual.html.) In doing so, a buried surface area is defined for each CDR.

### 2.2 The Dataset and Data Curation: The IMGT/3Dstructure-DB

We use the Ig–Ag complexes from the IMGT/3Dstructure-DB [http://www.imgt.org/3Dstructure-DB/ (20)], corresponding to the category *IG/Ag* for *IMGT complex type*. Each such complex is processed in order to identify *canonical complexes* involving one heavy chain, one light chain, and one ligand. Upon inspecting such cases, two decisions are made. First, on the antigen side, we retain three types only (peptide, protein, and chemical), due to the scarcity of cases involving other types. Moreover, we also remove complexes involving multiple ligands types. For the same reason, regarding species, complexes are assigned to three classes: human, mouse, and other. In total, 489 complexes are retained after filtering for missing data, inconsistencies, redundancy, ligand type, and species. The detailed processing methodology is described in the Section A.1 in Supplementary Material. The main features of the complexes used are also summarized in Table S3 in Supplementary Material.

CDR and FR limits of the VH and VL domains are according to the IMGT unique numbering (52) (Table S2 in Supplementary Material). Practically, we use the following notations: CDR1-IMGT of VH is written VH CDR1 and FR3-IMGT of VL is written VL FR3. Other CDRs and FRs follow the same scheme.

### 2.3 The Binding Affinity Benchmark

Our affinity predictions exploit the structure affinity benchmark (SAB) (23), a manually curated dataset containing 144 cases, each described by three crystal structures (of the unbound partners and of the complex) and the experimentally measured binding affinity in controlled conditions. In this work, we split the SAB into two sets: 14 Ig–Ag cases defining the test set (Table S3 in Supplementary Material) and 125 non-Ig–Ag cases defining the training set. Five complexes (among which 3 Ig–Ag) were removed from the SAB because only an upper bound on their *K_d_* was provided, or had too many missing atoms. Having learned a statistical model from the latter, we predict affinities for Ig–Ag complexes of the former. See details in the Supplementary Material section.

### 2.4 Predicting Ligand Types

Antigens in the dataset are categorized as chemical, peptide, and protein. Predicting the ligand type therefore requires to build a 3-class predictor.

#### 2.4.1 Relevant Variables

**Figure 1:**
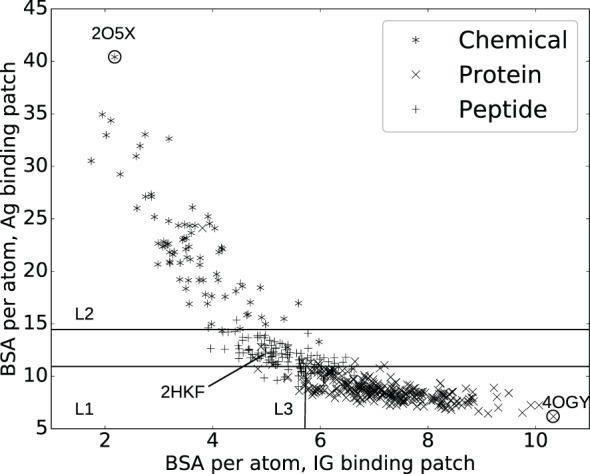
**Interaction specificity for Ig–Ag complexes: analysis and predictions**. Both analyses are based upon the average buried surface areas per atom [equations (2) and (3)] ${\bar{\text{bsa}}}_{\text{Ig}}$ versus ${\bar{\text{bsa}}}_{\text{Ag}}$. Scatter plot as a function of the ligand type. The three lines (L1, L2, and L3) show the partition defined by the decision tree, separating the ligand types (see main text). The points labeled 2O5X, 2HKF, and 4OGY correspond to complexes displayed in Figure S3 in Supplementary Material.

In order to predict ligand types, we represent each complex by two variables: ${\bar{\text{bsa}}}_{\text{Ig}}$ and ${\bar{\text{bsa}}}_{\text{Ag}}$ which are the average BSA per atom for atoms on the Ig and the Ag side, respectively. These variables define the two-dimensional space displayed in Figure 1 where each point represents a complex. A classifier, i.e., a method predicting the antigen type from the parameters ${\bar{\text{bsa}}}_{\text{Ag}}$ and ${\bar{\text{bsa}}}_{\text{Ig}}$ is then trained on this data. Practically, we use a decision tree partitioning the space into rectangular regions, each corresponding to a ligand type.

#### 2.4.2 Statistical Methodology

Since the performance of classifiers tested on the training data is overestimated and leads to classifiers with poor generalization abilities (overfitting), various schemes have been devised to obtain an estimate of the generalization error.

We use the *k*-fold cross-validation where the dataset is randomly divided in *k* subsets of equal size, and *k* − 1 subsets are alternatively used to classify the remaining one. At the end of this procedure, each sample has been predicted and the proportion of misclassified samples can be computed. Here, *k* is set to 5. Since the partition into training and test data used during this procedure is inherently random and may lead to non-representative results for a single run, we report median errors over 1,000 cross-validation runs.

In order to size the expected performance of a random classifier, we use a simple permutation test. Basically, complexes are randomly predicted by permuting the ligand types in the original dataset and assigning the result of the permutation to each complex. This procedure maintains the number of complexes per ligand type. Median errors over 10,000 random permutations are reported.

Finally, we also train a *naive* classifier which only uses the number of interface atoms. We report median errors over 1,000 cross-validation runs.

#### 2.4.3 Ligand Redundancy

In total, there are 465 distinct ligands out of 489 complexes, with the most represented ones appearing at most 3 times. Overfitting due to Ag redundancy in the dataset is therefore not an issue.

### 2.5 Predicting Binding Affinities

#### 2.5.1 Relevant Variables

The dissociation free energy is the thermodynamic quantity defined by equation (1). The estimation of −Δ*G* was recently revisited and posed as a sparse linear model estimation problem (24), stressing the importance of two variables. These two variables turn out to be the most informative ones when estimating binding affinities, in the sense where they get selected most often amidst a pool of variables modeling relevant biophysical properties (24).

The first one, the inverse volume-weighted internal path length (IVW-IPL), encodes the size and morphology of the interface and takes atomic packing into account. Let I be the set of interface atoms in a complex. Let SO(*a*) and Vol(*a*) be the shelling order and Vol_bound the volume of atom *a* in the complex (see section 2.1). The IVW-IPL is defined as follows:

(4) $$\text{IVW}-\text{IPL}=\underset{a\in I}{\sum }\;\frac{\text{SO}\left(a\right)}{\text{Vol\_bound}\left(a\right)}.$$

On the one hand, the shelling order refines so-called core–rim models (27). Borrowing to the notion of cooperative effects involving non-bonded weak interactions, an isotropic or disk-like interface is indeed expected to be more stable than an elongated one—even if their surface areas match. On the other hand, the atomic packing encodes the local density of neighbors of a given atom and thus provides a measure for local interactions (hydrogen bonds and van der Waals interactions). Note that packing is a subtle quantity related to the enthalpy–entropy compensation discussed in Introduction, as its properties strike a balance between enthalpy (a large number of neighbors favor interactions) and entropy (too small of a packing is detrimental for dynamics yielding an entropic penalty).

The second variable (NIS*^charged^*) is the fraction of charged residues on the non-interacting surface (NIS, i.e., the exposed surface of the Ig and of the Ag not involved in the interface). The NIS is meant to encode electrostatic properties and solvent interactions (30).

#### 2.5.2 Statistical Methodology

**Figure 2:**
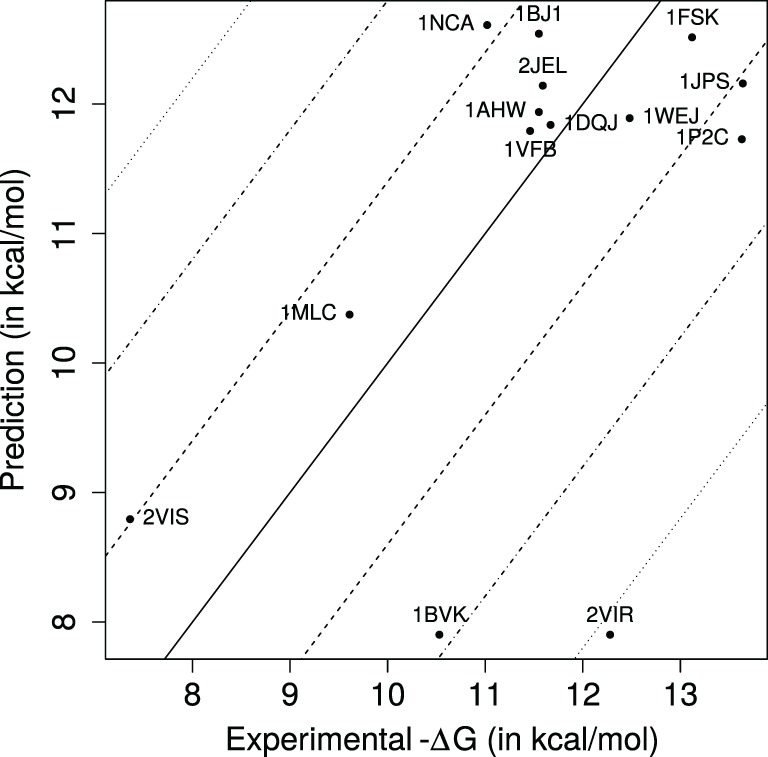
**Predicted versus experimental affinities for Ig–Ag complexes**. Dashed, dash-dotted, and dotted lines, respectively, show errors of ±1.4, ±2.8, ±4.2 kcal/mol, corresponding to *K_d_* approximated within one, two, and three orders of magnitude.

**Figure 3:**
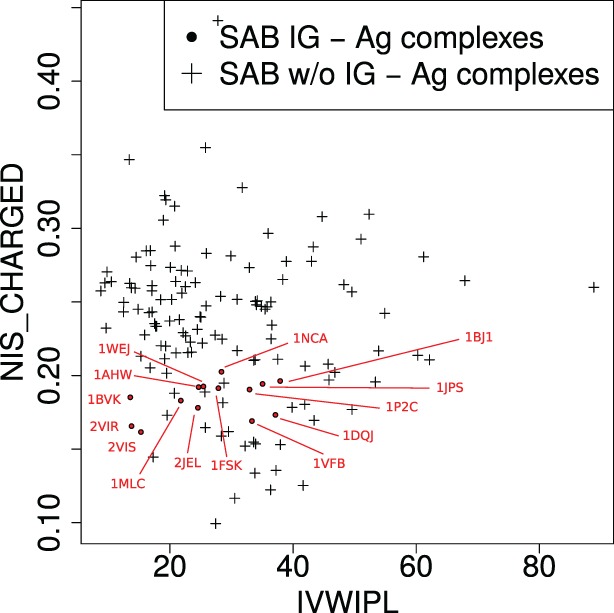
**Complexes in the two-parameter space of the model**. The model uses two variables (see main text): IVW-IPL, inverse volume-weighted internal path length; NIS_CHARGED, proportion of charged residues on the non-interacting solvent-accessible surface.

We compare experimental versus predicted binding affinities (Figure 2), the latter estimated using *k* nearest neighbors regression (knn) (53, 54), a non-parametric regression strategy which does not require any *a priori* on the mathematical model for the response variable estimated—as opposed to linear regression for instance. This strategy is a two step strategy. As a preprocessing step, we compute the parameters IVW-IPL and NIS*^charged^* for the training set (125 cases), yielding a point cloud *P* in the two-dimensional space defined by IVW-IPL and NIS*^charged^* (Figure 3). To estimate the affinity of a complex *q* (an Ig–Ag case), we proceed in two steps. First, the *k* nearest neighbors of *q* in *P* are sought, with *k* a predefined number. Second, the affinity of *q* is estimated by averaging those of its *k* nearest neighbors. [Practically, the scikit-learn library (55) was used, namely, the neighbors package for knn regression.]

**Figure 4:**
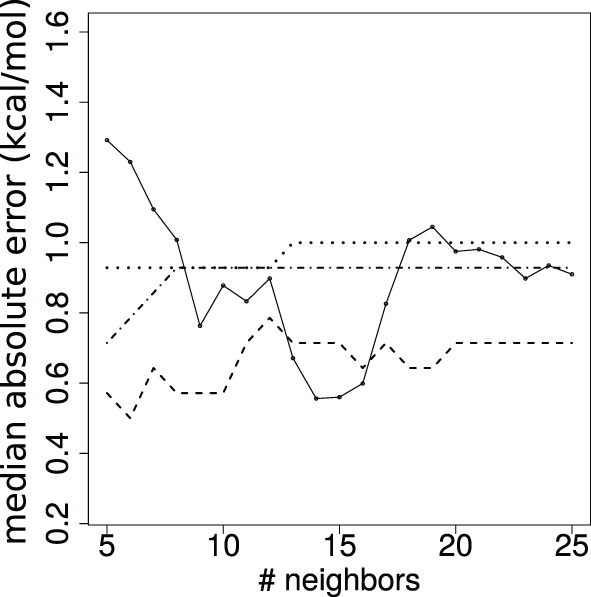
**Stability of affinity prediction**. Performance of the *k* nearest neighbors estimates when varying the number of neighbors *k*. Solid line: median absolute error (kcal/mol); dashed: dot-dashed; dotted lines: proportion of predictions with error below 1, 2, and 3 orders of magnitude, respectively.

We assess the quality of our predictions by varying the value *k*. From a theoretical standpoint (53), it is known that *k* must be super-logarithmic and sublinear in the number of cases processed. Since *log*(144) ~ 5, we explore the range *k* ∈ 5, …, 25 (Figure 4). The results discussed in the main text correspond to *k* = 10.

In order to assess the impact of the distance to nearest neighbors and of the consistency of their affinity values on the accuracy of the predictions, we compute the average distance *d_i_* between each Ig–Ag complex *i* and its *k* = 10 nearest neighbors in the training set (i.e., those used to estimate its binding affinity using k-nearest neighbor regression). We also compute the standard deviation of the affinity values σ*_i_* of these 10 nearest neighbors. These are compared to the absolute error |*e_i_*| (=|experimental_affinity*_i_* − predicted_affinity*_i_*|) of the prediction on complex *i*.

### 2.6 Comparing the Energetic Contribution of Interface Atoms between CDRs

To assess the respective energetic contributions of CDRs to binding affinity, we dissect the IVW-IPL [equation (4)] into the contributions of CDR1 + CDR2 and CDR3. We also compute the *average normalized shelling order* (or ANSO for short) for each CDR

(5) $$\text{ANSO}=\frac{1}{\left|A\right|}\underset{a\in A}{\sum }\;\frac{\text{SO}\left(a\right)}{\text{Vol\_bound}\left(a\right)},$$

with *A* is the set of interface atoms of the CDR and the size of this set is |A|. The distribution of IVW-IPL and ANSO between CDR1 + 2 and CDR3 within the same chain are then compared using a Wilcoxon signed-rank test.

## 3 Results

### 3.1 Characteristics of the Binding Patch Predict the Ligand Type

#### 3.1.1 Atomic Solvent Accessibility Asymmetry Is a Signature for the Ligand Type

A classical and informative variable describing a protein–protein interface is the buried surface area (BSA), which is known to correlate to the number of interface atoms (50). In our case, a Pearson coefficient equal to 0.99 is obtained. However, this value drops down to 0.82 and 0.89, respectively, for the Ig and the Ag sides, a fact owing to the shape complementarity between the binding patches on the Ig and Ag sides (Figure S4 in Supplementary Material). To further investigate this observation, we compute the average BSA per interface atom for both the Ig and Ag [equations (2) and (3)]. Strikingly, the ligand type has a strong impact on these quantities: complexes involving a chemical ligand have a higher average BSA per atom at the Ag side of the interface (${\bar{\text{bsa}}}_{\text{Ag}}$) than those involving a peptide ligand which in turn have a higher ${\bar{\text{bsa}}}_{\text{Ag}}$ than those involving a protein ligand (Figure 1). Note that ${\bar{\text{bsa}}}_{\text{Ag}}$ and ${\bar{\text{bsa}}}_{\text{Ig}}$ can be seen as proxies for curvature of the Ag and Ig binding patches, hence their strong inverse correlation due to the complementarity between binding patches on the Ig and Ag sides (Figures S3D–F in Supplementary Material). This inverse correlation is rather intuitive for small ligands but may not be trivial for bigger antigens. Our contribution corroborates this fact for a whole set of structures.

To further exploit the ability of the parameters ${\bar{\text{bsa}}}_{\text{Ag}}$ and ${\bar{\text{bsa}}}_{\text{Ig}}$ to characterize interfaces as a function of the ligand type, we build a decision tree classifier (Figure 1; Figure S5 in Supplementary Material).

The median cross-validated error over all classes is 9.6% over 1,000 repetitions whereas the permutation test resulted in a median error of 56%. In using the naive classifier (based upon the number of interface atoms, see section 2.4), the median error over 1,000 repetitions is 32%. More precisely, the median cross-validated error rates per class are 5%, 19%, and 7% for chemical, peptides, and proteins, respectively. The higher error rate for peptides is mostly due to the classifier predicting proteins instead of peptides (Table S4 in Supplementary Material), which is not unexpected as the criterion to classify polypeptides as peptides or proteins is not standardized. For comparison, the permutation test resulted in error rates of 84% for chemicals, 75% for peptides, and 41% for proteins; clearly showing the influence of the number of complexes per class on the accuracy of the prediction. As for the naive classifier (based upon the number of interface atoms), error rates of 29%, 100%, and 4% are obtained. Overall, our classifier is able to accurately predict ligand types, despite the fact that the data are unbalanced.

### 3.2 Binding Affinity Predictions

Our k-nearest neighbors-based model predicts 8 (57.14%), 13 (92.86%), and 13 of the dissociation constants *K_d_* within one, two, and three orders of magnitude, respectively, with a median absolute error of 0.878 kcal/mol, which corresponds in a ratio for *K_d_* equal to 4.4 (Figure 2). [We also note in passing that these estimates are consistent with those obtained with a cross-validated linear model (Table S6 in Supplementary Material).] In terms of correlation coefficients, one gets 0.488 (Pearson) and 0.291 (Spearman). These results are very good, as predicting *K_d_* within one order of magnitude is essentially the best one can hope for without modeling subtle effects such as the pH in particular (56). They are also informative from a biological standpoint, as an affinity enhancement of two orders of magnitude is typically observed during affinity maturation.

In order to compare these results to what could be expected from a null model, we take the average −Δ*G* of the training dataset (10.78 kcal/mol ±2.84) as prediction for all complexes. This results in a median absolute error of 1.03 kcal/mol, or equivalently, in a ratio for *K_d_* equal to 5.7. The previous conclusions must therefore be mitigated, since a simple null model can show good, albeit less so, performances as well.

In order to rationalize the varying accuracy of predictions depending on the complex, we compute the average distance *d_i_* between each Ig–Ag complex *i* and its 10 nearest neighbors in the training set. We also compute the standard deviation of the affinity values of these 10 nearest neighbors σ*_i_* (Figure S10 in Supplementary Material). Both *d_i_* and σ*_i_* are weakly correlated to the absolute prediction error $\left|e_{i}\right|$ with Pearson’s correlation coefficients of 0.57 and −0.57, respectively. Both coefficients are (weakly) significantly different from zero with p-values of 0.0312 and 0.03316, respectively. The correlation between |*e_i_*| and *d_i_*/σ*_i_* is higher, however, with a Pearson correlation coefficient equal to 0.72 and a p-value of 0.00363. This suggests that good binding affinity prediction can be obtained provided that sufficiently similar complexes are in the training set and that their affinity values are consistent with each other. Interestingly, this property also accounts for the good performances of the null model.

We now discuss a few cases, focusing on complexes with similar ligands (Table S3 in Supplementary Material).

PDB entry 2VIR consists of an influenza hemagglutinin in complex with a neutralizing Ig. PDB entry 2VIS is the same Ig in complex with an escape mutant of the previous hemagglutinin differing by only one mutation (T131I). This causes their −Δ*G* to differ by almost 5 kcal/mol which corresponds to a factor ~4,800 on *K_d_*. These two complexes lie very close to each other in the parameter space (Figure 3), and our model unsurprisingly predict similar affinity values. However, they are only accurate for 2VIS (Figure 2). This lack of accuracy may be due to the inability of our model to capture large energetic contribution from few residues—a difficulty calling for the identification of *hot spots* (57, 58)—or may be due to a deficient modeling of dynamical properties (38).

PDB entries 1BVK, 1DQJ, 1MLC, 1P2C, and 1VFB all share the same ligand (hen egg lysozyme C EC 3.2.1.17) with −Δ*G* ranging from 19.61 to 13.63 kcal/mol. Focusing on the V region of heavy chains, 1P2C and 1MLC are fairly similar with 90.5% identity; and 1BVK and 1VFB as well with 79.3% identity. All other pairs share limited sequence identity with values between 44.3 and 57.5%. For the V region of light chains, sequence identity between 1P2C, 1MLC, and 1DQJ ranges between 93.5 and 96.3%. Sequence identity between 1BVK and 1VFB is 82.2%. Sequence identity between other pairs ranges between 56.1 and 61.7%. PDB entries 1DQJ and 1VFB which have similar experimental affinities are located close to each other in the parameter space (Figure 3) and are both well predicted by our model (Figure 2). PDB entry 1MLC is also well predicted even though its experimental affinity is much lower than that of the previous entries. On the other hand, 1BVK and 1P2C are not so well predicted with errors between one and two orders of magnitude on *K_d_*, although the latter is located in the same region as 1DQJ and 1VFB. Interestingly, both are underestimated as is the case for 2VIR and 2VIS, which could come from the aforementioned limitations of our model. Finally, Igs whose V regions have similar sequences do not necessarily have similar binding affinity for the same antigen.

The success of the affinity prediction owes to two important properties of the learning set (non-Ig–Ag complexes) and the training set (Ig–Ag complexes). First, Ig–Ag complexes fall in a reduced region of the space defined by the two parameters IVW-IPL and NIS*^charged^* of the model, i.e., they are similar from the point of view of the model. Second, the Ig–Ag complexes fall in a region which is well represented in the training set (i.e., the rest of the SAB). This means that in the space of the two parameters of the model, Ig–Ag complexes are similar to the other protein–protein complexes of the SAB. In order to predict the binding affinity of Ig–Ag complexes with protein ligands, our model therefore takes advantage of the fact that they are similar both to each other and similar to other protein–protein complexes.

#### 3.2.1 Comparison with the PRODIGY Server

In order to see how our approach fares against the state of the art, we compare our results against the PRODIGY server. The PRODIGY server is one of the most recent tools for affinity prediction (59) and is based on the work from Vangone and Bonvin (60).

The accuracy of PRODIGY is lower than that of the current study with median absolute errors of 1.4 versus 0.878 kcal/mol, respectively. For reference, we also provide the root mean squared errors (2.226 versus 1.676 kcal/mol), Pearson’s correlation coefficients (0.149 versus 0.488), and Spearman’s correlation coefficients (0.238 versus 0.291). Interestingly, our method is successful at predicting similar affinities (Figure S10 in Supplementary Material) for five complexes (1AHW, 1DQJ, 1VFB, 2JEL, and 1BJ1) for which PRODIGY predicts widely varying values.

#### 3.2.2 CDRs: Lengths and BSA

It has been observed that CDR lengths differ between different antigen types (17, 61), a finding suggesting that CDR lengths influence the binding site to accommodate the ligand. We therefore undertook the characterization of this relationship in the IMGT/3Dstructure-DB. Since all the atoms of a CDR may not contribute to the interface, we investigate the correlation between the length of a CDR and its contribution to the BSA. As CDR1 and CDR2 are both encoded by V genes, we study them together and subsequently investigate the relationship between [CDR1⋅CDR2] pairs and BSA on the one hand, and CDR3 and BSA on the other hand. We observe that CDRs of a given length can display widely varying levels of BSA (Figures S6 and S7 in Supplementary Material). These results indicate that CDR lengths must be complemented to fully describe the involvement of a CDR in the interaction with the Ag. This is backed up by the very limited ability of neural networks trained on sequence data only to predict the ligand type bound by an Ig in Ref. (61). An error rate of 54% is indeed observed, to be compared to a baseline of 75% for a random predictor on four classes (protein, hapten, nucleotide, and viral protein) (61).

#### 3.2.3 Respective Contributions of the CDRs to the Interface, for VH and VL Domains

**Figure 5:**
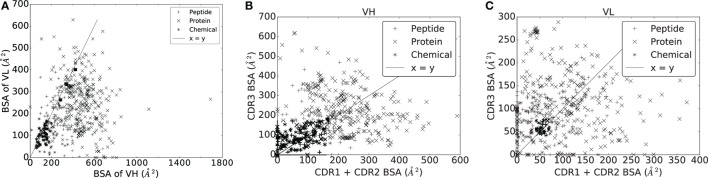
**Buried surface area (*A*^2^) of the VH and VL domains, and their respective CDRs**. **(A)** VH versus VL. **(B)** VH domain. **(C)** VL domain.

In an Ig–Ag complex, it is generally believed that VH contributes more to the recognition than VL. With a BSA of VH strictly larger than that of VL for 430/489 complexes (~86%) (Figure 5A), our analyses support this idea. To refine this view, we split the BSA into contributions by the CDRs within a V domain, observing a great deal of variation across the dataset, independent from the ligand type (Figures 5B,C). A general observation is that the sum of contributions of CDR1 and CDR2 essentially matches that of CDR3 for both VH and VL. Consider the sum of the BSA of CDR1 and CDR2 on one hand, and the BSA of CDR3 on the other hand. The first quantity is larger than the second one for ~46% of the complexes for VH and for ~40% of the complexes for VL. Moreover, a Wilcoxon signed-rank test does not find a significant difference between them for VH (two-sided p-value = 0.1460) but does for VL (two-sided p-value = 0.0001), indicating that the contribution of CDR3 in terms of BSA and relative to other CDRs from the same chain is higher for the light chain than for the heavy chain.

**Figure 6:**
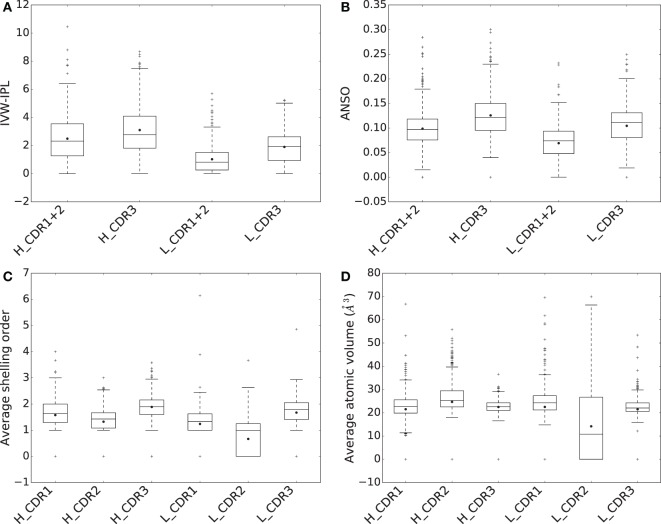
**Comparison of CDRs in terms of (A) inverse volume-weighted internal path length (IVW-IPL), (B) average normalized shelling order (ANSO), (C) average shelling order, and (D) average atomic volumes**.

To assess the contributions of CDRs to binding energy, we compute both their IVW-IPL and ANSO [equations (4) and (5)] for all complexes (Figures 6A,B). We then compare the distributions of these two quantities for CDR1 + 2 and CDR3 in the same chain, using a Wilcoxon signed-rank test at significance level α = 0.01. Consider the sum of the IVW-IPL of CDR1 and CDR2 on one hand, and the IVW-IPL of CDR3 on the other hand. The first quantity is larger than the second one for ~41% of the complexes for VH and for ~27% of the complexes VL (Figure S8 in Supplementary Material). Wilcoxon signed-rank tests find significant differences between them for both VH (two-sided p-value = 6.404 × 10^−7^) and VL (two-sided p-value = 7.217 × 10^−30^). Removing the dependence on the number of atoms, i.e., comparing the ANSO distribution computed on both CDR1 and CDR2 on the one hand and CDR3 on the other hand, leads to significant differences as well for VH (two-sided p-value = 6.221 × 10^−30^) and VL (two-sided p-value = 2.480 × 10^−37^).

Thus, as opposed to the results obtained when considering the BSA, the sum of contributions to the binding affinity of CDR1 and CDR2 is significantly lower than that of CDR3 for both VH and VL.

For both chains, the difference in ANSO can be imputed to two facts. First the average shelling order (Section 2.1) for atoms of the CDR3 is higher than those of CDR1 and 2 (Figure 6C). Second, their average atomic volume is lower (Figure 6D). Both are related since the shelling order and the atomic volume are negatively correlated (Figure S8C in Supplementary Material).

## 4 Discussion

In this work, we provide a precise quantitative description of Ig–Ag interfaces, leading to an accurate classification of ligand types and to accurate binding affinity predictions. We also quantify the contributions made by CDRs at interface both in terms of surface area and binding energy, and we show that VH CDR3 is the main factor determining binding affinity and interaction specificity. While these facts were previously known from a qualitative standpoint, the task of designing quantitative models supporting them had remained elusive, with insights focused on specific conformations. Instead, our models provide quantitative estimates illustrating the relationship between structure, dynamics, and affinity of Ig–Ag complexes.

### 4.1 Enhanced Specificity and Affinity Descriptions from Global Interface Statistics

The buried surface area (BSA) of a protein complex has long been known to be a simple and informative descriptor of interfaces (46). We refine this statistic by computing the average BSA contributed by interfacial atoms from the Ig (statistic ${\bar{\text{bsa}}}_{\text{Ig}}$) and the Ag (statistic ${\bar{\text{bsa}}}_{\text{Ag}}$). These quantities turn out to be clear a signature of the ligand type, a property which can further be exploited for classification purposes. While the classification of Ig–Ag interfaces into classes depending on structural features has already been addressed (62, 63), our parameters are the first ones yielding such a clear separation between specific antigen types.

To complement this analysis, we perform binding affinity predictions for 14 Ig–Ag complexes, based on structural parameters encoding enthalpic and entropic quantities (24). Our predictions of *K_d_* are accurate within two orders of magnitude for all but one complex and within one order of magnitude for 8 of them. Interestingly, these results stress the relevance of the overall approach, which exploits structural and functional similarities between the test set (the Ig–Ag complexes) and the training set (the SAB deprived from the Ig–Ag complexes). In fact, the high accuracy of our predictions shows that the binding affinity prediction problem could be partially solved using large databases of Ig–Ag complexes with binding affinity measurements.

Our results on specificity analysis and affinity predictions are of immediate practical relevance in the context of Ig design and Ig–Ag docking. Docking is the problem of predicting the pose (i.e., the static structure) and the affinity of a complex from the unbound partners (64). The latter problem is harder than the former, another embodiment of the role of dynamics in the emergence of function. Our parameters are of high interest for both problems. At the pose prediction stage, they provide filters to check that putative Ig–Ag complexes proposed by docking algorithms comply with our classification rules, as a function of the ligand type. In a similar spirit, these parameters are of direct relevance to predict the ligand type from the structure of the Ig VH + VL domains. At the affinity prediction stage, assuming a good quality (i.e., resolution) putative structure for the complex, reliable affinity predictions can be made.

These results also call for extensions, in particular to handle different ligand types (peptides and haptens). Since the quality of predictions owes in particular to a good coverage of the region of the model space targeted by predictions, this extension is likely to be successful assuming a database—identical in spirit to the SAB, providing sufficiently many cases to learn from. From a formal standpoint, we also envision progress on the analysis of the correctness of affinity predictions, based on two ingredients. The first one is the accuracy of estimators for thermodynamic quantities, using parameters such as those used in this work. The second one is the mathematical convergence of regressors, in particular those based on nearest neighbors, as used in this work.

### 4.2 Bridging the Gap between Structure, Dynamics, and Function

Our findings show that global structural parameters perform remarkably well to predict affinity and specificity, which are notions formally defined in the realm of thermodynamics. It is therefore instrumental to understand which features of CDRs explain the relevance of our parameters. In other words, it appears important to consider at once the role of the six CDRs for most antibody specificities.

If the molecules studied were perfectly rigid, local interactions (hydrogen bonds and van der Waals interactions) would play a prominent role in the formation of the Ig–Ag complex, and the comparable BSA contributed by CDR1 + 2 vs CDR3 would hint at commensurable contributions from all CDRs. This purely enthalpic view is, however, insufficient, as preconfiguration/prerigidification of the binding site may yield a decreased entropic loss upon complex formation, hence an enhanced binding affinity (35–38). A useful proxy for dynamics is the length of VH CDR3, and difficulties were observed to define canonical conformations for VH CDR3 (3, 5, 6, 8, 10, 13) as opposed to the other CDRs. Indeed, accurate sequence-based conformation predictions are limited to the base or *torso* of the VH CDR3. In this work, we code the enthalpy–entropy compensation (see discussion in section 2.5) using packing properties via our parameters IVW-IPL and ANSO. This leads to two important observations: first, independently of the number of interface atoms, VH CDR3 contributes significantly more to the binding energy than VH CDR1 and VH CDR2 combined; second, interface atoms in VH CDR3 are more closely packed than in other CDRs in the heavy chain. The latter point implies that it is important to minimize the entropic penalty entailed upon binding, which can be achieved by preformation, i.e., the CDR is in bound conformation prior to the binding event. Interestingly, the authors of Ref. (18) come to the conclusion that VH CDR3 is responsible for the specificity of the interaction whereas the other CDRs account for its stability. We provide a quantitative view on this property, based on our parameters IVW-IPL and ANSO.

Summarizing, the genetic variability of VH CDR3 is complemented structurally by its dynamic nature to make it the main factor involved in the determination of the specificity and increase of affinity of an Ig for an Ag. It should be stressed that, although this observation can be used as a guide during the design of Ig, it is by no means necessary, as tight binders can be designed *de novo* without any CDR—see Ref. (65) for an example involving the stem of influenza virus hemagglutinin.

Naturally, one should also expand our analysis at the whole Ig level, as various structural features of Igs influence their efficacy in the immune response. These include the ball-and-socket joint relating VL and VH, the CL and CH1 constant domains (66, 67), and more generally the constant regions which have been shown to influence the avidity (41–44), and are involved in Ig effector properties, such as ADCC or CDC (68). A quantitative assessment of the role of these features requires going beyond the Ig–Ag interface level, with a clear focus on the dynamics of the whole Ig protein. Again, the identification of the most relevant degrees of freedom in such regions may pave the way to efficient simulation and design strategies.

## Author Contributions

FC and SM designed and conducted the research and wrote the paper. PB and M-PL wrote the paper.

## Conflict of Interest Statement

The authors declare that the research was conducted in the absence of any commercial or financial relationships that could be construed as a potential conflict of interest.

## Abbreviations

SAB, structure affinity benchmark; SAM, solvent-accessible model; IG, immunoglobulin; Ag, antigen; CDR, complementarity determining region; FR, framework region.
