# Supplementary material for: Novel Structural Parameters of Ig–Ag Complexes Yield a Quantitative Description of Interaction Specificity and Binding Affinity
Source: Front Immunol. 2017 Feb 9;8:34. doi: 10.3389/fimmu.2017.00034 (PMC5298999; doi:10.3389/fimmu.2017.00034)
Supplement: Supplementary file 1 [file Presentation_1.PDF]

A Supplementary information

A.1 The IMGT dataset

A.1.1 Ig - Ag complexes

We use the Ig - Ag complexes from the IMGT/3Dstructure-DB (`http://www.imgt.org/3Dstructure-DB/` [1]), corresponding to the category *IG/Ag* for *IMGT complex type*. Only IMGT-PDB files are kept.

This dataset features 1602 files. Each such complex is processed in order to identify canonical complexes involving one heavy chain, one light chain, and one ligand (Section SA.1.2). A total of 1275 canonical complexes are thus extracted, of which 554 non-redundant complexes. After further filtering on the ligand types (keeping only complexes with a single ligand type among protein, peptide and chemical), 489 complexes remain.

Fig. S 1 Size of the antigens (number of atoms) Two large peptides (IMGT-PDB file 3W11 chain E, 2301 atoms, and IMGT-PDB file 4R4N chain I, 5172 atoms) are not displayed for readability. See section 2.2 for the data curation methods.

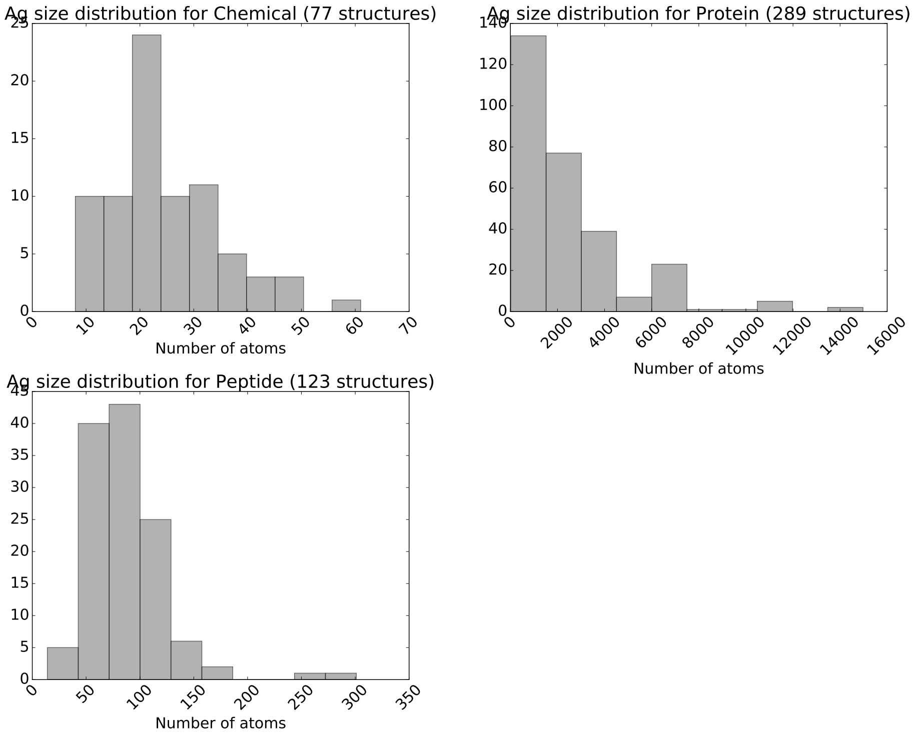

Table S I Summary of the number of Ig - Ag complexes in each class of species / ligand type. The dataset includes VH (V-domains of heavy chains)and VL comprising V-KAPPA (V domains of kappa chains) and V-LAMBDA (V domains of lambda chains. See section 2.2 for the data curation methods.

	Mouse	Human	Other	total
Peptide	80	32	11	123
Protein	168	91	30	289
Chemical	65	7	5	77
total	313	130	46	489

Table S II Amino acid positions associated with each IMGT label defining the decomposition of a V-domain into seven regions Positions of the complementarity determining regions (CDRs) using the IMGT numbering scheme [2]. See section 2.2 for the data curation methods.

**Region**	FR1	CDR1	FR2	CDR2	FR3	CDR3	FR4
**start-stop**	1 - 26	27 - 38	39 - 55	56 - 65	66 - 104	105 - 117	118 - 128

A.1.2 Inferring canonical complexes

Canonical complexes. A canonical configuration for a IMGT/3Dstructure-DB IMGT-PDB file is as follows: *one H chain, one L chain, one ligand.* A non canonical configuration may occur for different reasons:

- The asymmetric unit of the crystal structure contains two or more Fabs.
- Several molecules have co-cristallized with the Ig - Ag complex.
- Two Ig chains, H and L, and one Ag chain are found but the Ig chains are not annotated as forming a receptor in the IMGT 410 section.
- An Ig receptor is annotated as containing more than two chains.
- The ligand is a multi-chain protein

The following issues are faced:

- A file may not be canonical *i.e.* there might be several complexes in a single file.
- There might be some issues with the numbering of the chains.
- There might be missing data (residues, chains information, labels)
- Several complexes might be similar and bias the results.
- Some molecules annotated as ligand may actually be buffer molecules (*e.g.* glycerol)
- Some purification proteins remain (*e.g.* protein L, A or G) and do not engage in specific contacts with the Ig

Using the executable `sbl-intervor-ABW-atomic.exe` from the structural bioinformatics library (SBL, `sbl.inria.fr`), which implements the Voronoi interface model presented in section 2.1, we proceed in two steps. First, we infer the chains *pairings* in every file which does not contain a canonical complex. For this, we compute the interfaces between all pairs of chains. We then group L and H chains in pairs for which the number of atoms at the interface is the highest. We then assign the ligand(s) chains to the HL pairs if they make contacts with either chain.

Note that in the case where an Ag is in contact with several Ig, it will be assigned to both Ig.

Finally, all buffer molecules and Ig purification proteins (namely protein L, A and G) whose annotated name satisfy the regexp `"immunoglobulin g-binding | protein[ ]+[gl]($|\s|\’) | glycerol | 2-Amino-2-Hydroxymethyl-Propane-1,3-Diol | tris | 2-(N-Morpholino)-Ethanesulfonic Acid"` are removed from the files because they are not representative of Ig - Ag interactions.

Crystal contacts. The previous automatic detection raises the problem of crystal contacts, since complexes reported might be false positives.

They could potentially be ruled out by using a cutoff such as the minimal number of atoms at an interface to be considered significant, however, there might also be few contacts between a Fab and a small ligand. It would therefore be necessary to study the distribution of the number of atoms at the interface for different classes of ligands to set a specific cutoff.

To circumvent this issue, we currently exclude from the analysis complexes in which the ligand does not make at least one contact with the variable domain (CDR or FR).

A.1.3 Removing redundancies from IMGT/3Dstructure-DB

Redundant complexes may come from two sources: the same complex may be found in the same asymmetric crystal unit, or it may be found in two different IMGT-PDB files.

We therefore need to remove the redundancy of the dataset to avoid biasing the statistics. For this, we need to consider similarities at the interface level. Once all complexes are extracted from the database, we need to compare the interfaces of all pairs of complexes, group complexes having a similar interface, and keep one representative complex for each group.

We rely on a quick method based upon IMGT labels. Consider triplets formed by the IMGT labels of both Ig chains and the Ag chain (*e.g.* `(VH-CH1, L-KAPPA, Capsid protein C)`). We record triplets which have already been included in the analysis and exclude complexes which have the same triplet.

A.2 The binding affinity benchmark dataset

Test set. The SAB contains 17 Ig - Ag cases (PDB IDs: 1AHW, 1BJ1, 1BVK, 1DQJ, 1E6J, 1FSK, 1IQD, 1JPS, 1MLC, 1NCA, 1NSN, 1P2C, 1VFB, 1WEJ, 2JEL, 2VIR and 2VIS). Their K_d was determined at temperatures ranging between 20 and 25 °C or reported as ambient/room temperature. The temperature was not reported in one case. The pH during measurements ranged between 7 and 7.5 except in one case where it was 4.8 (1BJ1). It was not reported in five cases, and for two it is likely to have been 7.4 (BIAcore standard). All the Igs are either murine or

humanized monoclonal Igs raised against their antigen *in vivo* or *in vitro*, with K_d ranging from $4 \cdot 10^{-6}$ to 10^{-10} kcal/mol (or equivalently, -ΔG ranging from 7.36 to 13.64 kcal/mol). Out of these 17 cases, 1IQD and 1NSN are discarded as only an upper bound on their K_d is provided in the SAB. Furthermore, 1E6J is also discarded because too many atoms could not be matched between the bound and unbound structures. The 14 remaining cases only involve protein ligands. Among them, five are hen egg lysozymes (HEL), two are a tissue factors(TF), two are hemagglutinins (HA), and the remaining ones are birch pollen allergen (Bet v 1), cytochrome c (Cytc), HPr protein, neuraminidase (NA) and vascular endothelial growth factor (VEGF). We note that the iRMSD and the total RMSD between the bound and unbound form of the Igs are always smaller than 1.24Å and 0.95Å respectively. That is, the 14 cases are essentially rigid cases.

Training set. The rest of the SAB is used to train the model and is called *training set* in the sequel. 1ZLI is removed from the training set because too many atoms could not be matched between the bound and unbound structures and 1UUG is also removed because only an upper bound on its K_d is provided.

Table S III Main features of the Ig - Ag complexes found in the structure affinity benchmark.

CDR bounds correspond to the first and last residue numbers in IMGT renumbered PDB files.

PDB ID	Ig H chain	Ig L chain	Ag chain(s)	Ag type	Species	VH V and J gene	VL V and J gene
1AHW	B	A	C	Protein	Mus musculus (house mouse)	IGHV14-1*02 IGHJ2*01	IGKV14-111*01 IGKJ2*01
1BJ1	H	L	WV	Protein	Humanized (humanized)	IGHV7-4-1*02 IGHJ2*01	IGKV1-33*01 IGKJ1*01
1BVK	E	D	F	Protein	Humanized (humanized)	IGHV4-59*01 IGHJ4*03	IGKV1-27*01 IGKJ1*01
1DQJ	B	A	C	Protein	Mus musculus (house mouse)	IGHV3-8*02 IGHJ6*03	IGKV5-43*01 IGKJ1*02
1FSK	C	B	A	Protein	Mus musculus (house mouse)	IGHV1-61*01 IGHJ3*01	IGKV6-20*01 IGKJ1*02
1JPS	H	L	T	Protein	Homo sapiens (human)	IGHV3-66*04 IGHJ4*03	IGKV1-39*01 IGKJ1*01
1MLC	B	A	E	Protein	Mus musculus (house mouse)	IGHV1-9*01 IGHJ2*01	IGKV5-43*01 IGKJ2*01
1NCA	H	L	N	Protein	Mus musculus (house mouse)	IGHV9-3*03 IGHJ2*01	IGKV6-25*01 IGKJ1*01
1P2C	B	A	C	Protein	Mus musculus (house mouse)	IGHV1-9*01 IGHJ4*01	IGKV5-43*01 IGKJ1*01
1VFB	B	A	C	Protein	Mus musculus (house mouse)	IGHV2-6-7*01 IGHJ2*01	IGKV12-41*02 IGKJ2*01
1WEJ	H	L	F	Protein	Mus musculus (house mouse)	IGHV14-3*02 IGHJ2*01	IGKV12-41*02 IGKJ1*01
2JEL	H	L	P	Protein	Mus musculus (house mouse)	IGHV1-67*01 IGHJ1*01	IGKV1-117*01 IGKJ1*02
2VIR	B	A	C	Protein	Mus musculus (house mouse)	IGHV2-9*02 IGHJ4*01	IGLV1*01 IGLJ1*01
2VIS	B	A	C	Protein	Mus musculus (house mouse)	IGHV2-9*02 IGHJ4*01	IGLV1*01 IGLJ1*01

PDB ID	VH CDR lengths (1, 2, 3)	VL CDR lengths (1, 2, 3)	Ag size (number of atoms)	Ag name
1AHW	8 8 10	6 3 9	1612	Thromboplastin (synonym: tissue factor, TF, coagulation factor
1BJ1	8 8 16	6 3 9	1522	VEGF (Vascular endothelial growth factor A)
1BVK	8 7 10	6 3 9	1001	Lysozyme C [hen egg white] (HEL) EC:3.2.1.17
1DQJ	8 7 7	6 3 9	1007	Lysozyme C [hen egg white] (HEL) EC:3.2.1.17
1FSK	8 8 11	6 3 9	1230	Major birch pollen allergen Bet v1
1JPS	8 8 10	6 3 9	1611	Tissue Factor
1MLC	8 8 9	6 3 9	1001	Lysozyme C [hen egg white] (HEL) EC:3.2.1.17
1NCA	8 8 13	6 3 9	3075	Neuraminidase [influenza virus, A/Tern strain, N9 subtype]
1P2C	8 8 9	6 3 9	1001	Lysozyme C [hen egg white] (HEL) EC:3.2.1.17
1VFB	8 7 10	6 3 9	1265	Lysozyme C [hen egg white] (HEL) EC:3.2.1.17
1WEJ	8 8 10	6 3 9	826	Cytochrome c [horse]
2JEL	8 8 11	11 3 9	640	Histidine-containing protein of the phosphoenolpyruvate: sugar
2VIR	8 7 16	9 3 9	2075	Hemagglutinin HA1 [influenza virus]; residues: 28-328
2VIS	8 7 16	9 3 9	2076	Hemagglutinin HA1 [influenza virus] T131I (escape mutant);

PDB ID	Ig name	Resolution	VH CDR1 bounds	VH CDR2 bounds	VH CDR3 bounds	VL CDR1 bounds	VL CDR2 bounds	VL CDR3 bounds
1AHW	AB-GAMMA-1_KAPPA	3.0	27 38	56 65	105 117	27 38	56 65	105 117
1BJ1	AB-GAMMA-1_KAPPA	2.4	27 38	56 65	105 117	27 38	56 65	105 117
1BVK	V-HEAVY_KAPPA	2.7	27 38	56 65	105 117	27 38	56 65	105 117
1DQJ	AB-GAMMA-2A_KAPPA	2.0	27 38	56 65	105 117	27 38	56 65	105 117
1FSK	AB-GAMMA-1_KAPPA	2.9	27 38	56 65	105 117	27 38	56 65	105 117
1JPS	AB-GAMMA-1_KAPPA	1.85	27 38	56 65	105 117	27 38	56 65	105 117
1MLC	FAB-GAMMA-1_KAPPA	2.5	27 38	56 65	105 117	27 38	56 65	105 117
1NCA	AB-GAMMA-2A_KAPPA	2.5	27 38	56 65	105 117	27 38	56 65	105 117
1P2C	FAB-GAMMA-1_KAPPA	2.0	27 38	56 65	105 117	27 38	56 65	105 117
1VFB	FV-HEAVY_KAPPA	1.8	27 38	56 65	105 117	27 38	56 65	105 117
1WEJ	AB-GAMMA-1_KAPPA	1.8	27 38	56 65	105 117	27 38	56 65	105 117
2JEL	AB-GAMMA-1_KAPPA	2.5	27 38	56 65	105 117	27 38	56 65	105 117
2VIR	AB-GAMMA-1_LAMBDA	3.25	27 38	56 65	105 117	27 38	56 65	105 117
2VIS	AB-GAMMA-1_LAMBDA	3.25	27 38	56 65	105 117	27 38	56 65	105 117

PDB ID	K_d (M)	-ΔG (kcal/mol)	iRMSD (Å)	Method	pH
1AHW	$3.40\cdot10^{-9}$	11.55	0.69	Competitive Inhibition assay	not stated
1BJ1	$3.40\cdot10^{-9}$	11.55	0.5	SPR	4.8
1BVK	$1.40\cdot10^{-8}$	10.53	1.24	Stopped-flow inhibition	7
1DQJ	$2.80\cdot10^{-9}$	11.67	0.75	SPR	7.5
1FSK	$2.40\cdot10^{-10}$	13.12	0.45	SPR	7.4
1JPS	$1.00\cdot10^{-10}$	13.64	0.51	SPR	7.2
1MLC	$9.10\cdot10^{-8}$	9.61	0.6	SPR	7.4
1NCA	$8.30\cdot10^{-9}$	11.02	0.24	Fluorescence inhibition assay	7.2
1P2C	$1.02\cdot10^{-10}$	13.63	0.46	SPR	not stated
1VFB	$3.70\cdot10^{-9}$	11.46	1.02	ITC	7.1
1WEJ	$7.14\cdot10^{-10}$	12.48	0.31	Spectroscopic inhibition assay	not stated
2JEL	$2.80\cdot10^{-9}$	11.59	0.17	Fluorescence inhibition assay	7.2
2VIR	$1.00\cdot10^{-9}$	12.28	0.8	SPR	not stated (BIAcore standard:7.4)
2VIS	$4.00\cdot10^{-6}$	7.36	0.8	SPR	not stated (BIAcore standard: 7.4)

A.3 Hierarchical Voronoi interface models

Consider a complex where partner A is an Ig, and partner B an antigen. We wish to accommodate the hierarchical structure of the Fab [3]. We focus on the variable domains of the heavy and light chains, denoted VH and VL respectively, and decompose each of them into seven regions, namely three Complementarity Determining Regions (CDRs), and the four Framework Regions (FRs) flanking them [2] (Table SII). For example, a V domain is decomposed as FR1 + CDR1 + FR2 + CDR2 + FR3 + CDR3 + FR4.

Consider the partition of the variable domains VH and VL induced by the previous 14 labels. For the sake of conciseness and since we focus on interfaces involving the variable domains only, the domains VH and VL are plainly denoted H and L. Using these notations, we partition the $IGAg$ interface as follows:

- Hierarchical bicolor interface (no water): $IGAg = (L \cup H)Ag = LAg \cup HAg$
- Hierarchical mediated interface (water mediated only): $IGW - AgW = (LW - AgW) \cup (HW - AgW)$
- Hierarchical tricolor interface (both): $IGAgW = IGAg \cup (IGW - AgW)$

Analogously, the partition of the H (or L) V-domain into seven CDR and FR regions induces a partition of the HAg (or LAg) interface (Fig. S2).

The Voronoi facets associated to pairs of type (A,B) define the *bicolor* interface $A - B$ (bicolor since there are two partners); those associated to pairs of type (A,W) and (B,W) define the mediated interface $AW - BW$, since interactions between A and B are mediated by W(ater) molecules; finally, the union of the bicolor and mediated interface define the *tricolor interface* ABW. Geometrically, this interface is a polyhedron separating the partners. The curvature of this polyhedron is easily computed [4], and has been shown to provide information on binding modes [5].

Fig. S 2 Decomposition of an Ig - Ag complex. The Ig (or the Fab fragment) is decomposed into heavy (H) and light (L) chains (one H and one L per Fab) whose variable domains only (VH and VL) are of interest in this study. These domains are further decomposed into three complementarity determining regions (CDRs) and four framework regions (FRs). The Voronoi interface of Fig. S3 is partitioned into contributions from these 14 regions.

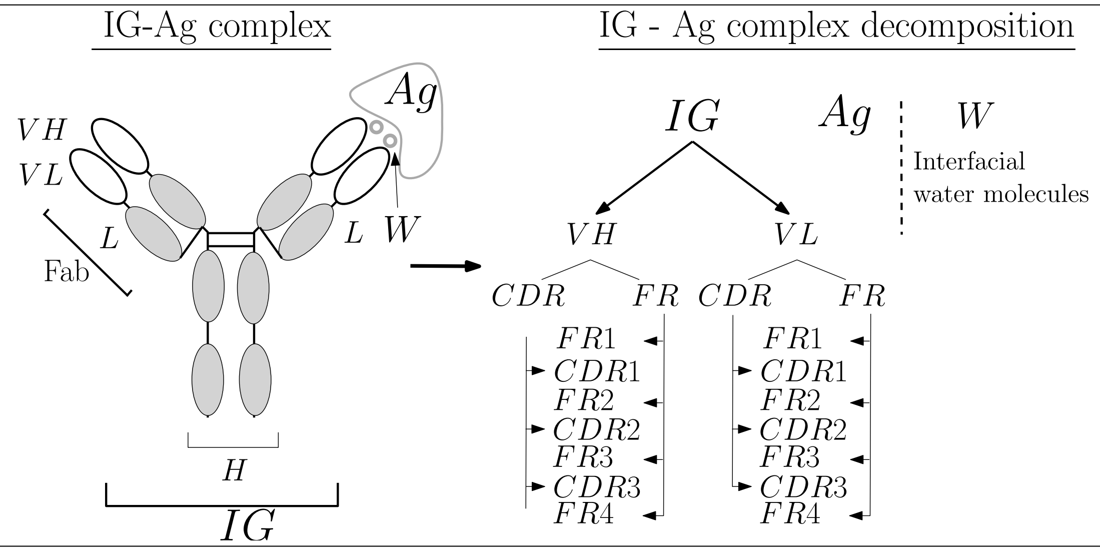

Fig. S 3 Voronoi interface model of an Immunoglobulin - Antigen (Ig - Ag) complex, defined from the solvent accessible model of the crystallographic complex. The Ig consists of H and L chains, with here the VH and VL domains shown in grey (cartoon representation), while the Ag consists of the chain in blue (CPK representation). **(A)** Ig - Ag complex, with the six complementarity determining regions (CDRs) colored using the IMGT conventions (VH CDR1: red, VH CDR2: orange, VH CDR3: purple, VL CDR1: blue, VL CDR2: green, VL CDR3: green-blue). **(B)** The Voronoi interface is a polyedral model separating the partners, whose parameters (area, curvature) convey information about the binding modes. **(C)** Each face of the Voronoi interface involves two interacting atoms, either from the partners or the interfacial water molecules sandwiched between them. The *buried surface area* (BSA) on each partner (by the second partner and interfacial water) is of prime interest to describe the interface. For the Ig, the BSA can be charged to the CDRs and framework regions (FRs). **(C, inset)** The interface atoms of a partner define its binding patch, which can be shelled into concentric shells (from the outside to the core), defining a distance to the patch boundary. The binding patch on the Ig side is shown from above (inset) where purple, blue and cyan identify atoms with shelling order 1, 2 and 3 respectively. **(D, E, F)** Voronoi interface of three complexes in (a) to illustrate different types: convex on the Ig side (small chemical ligand), saddle-like (peptide ligand), concave on the Ig side (protein ligand).

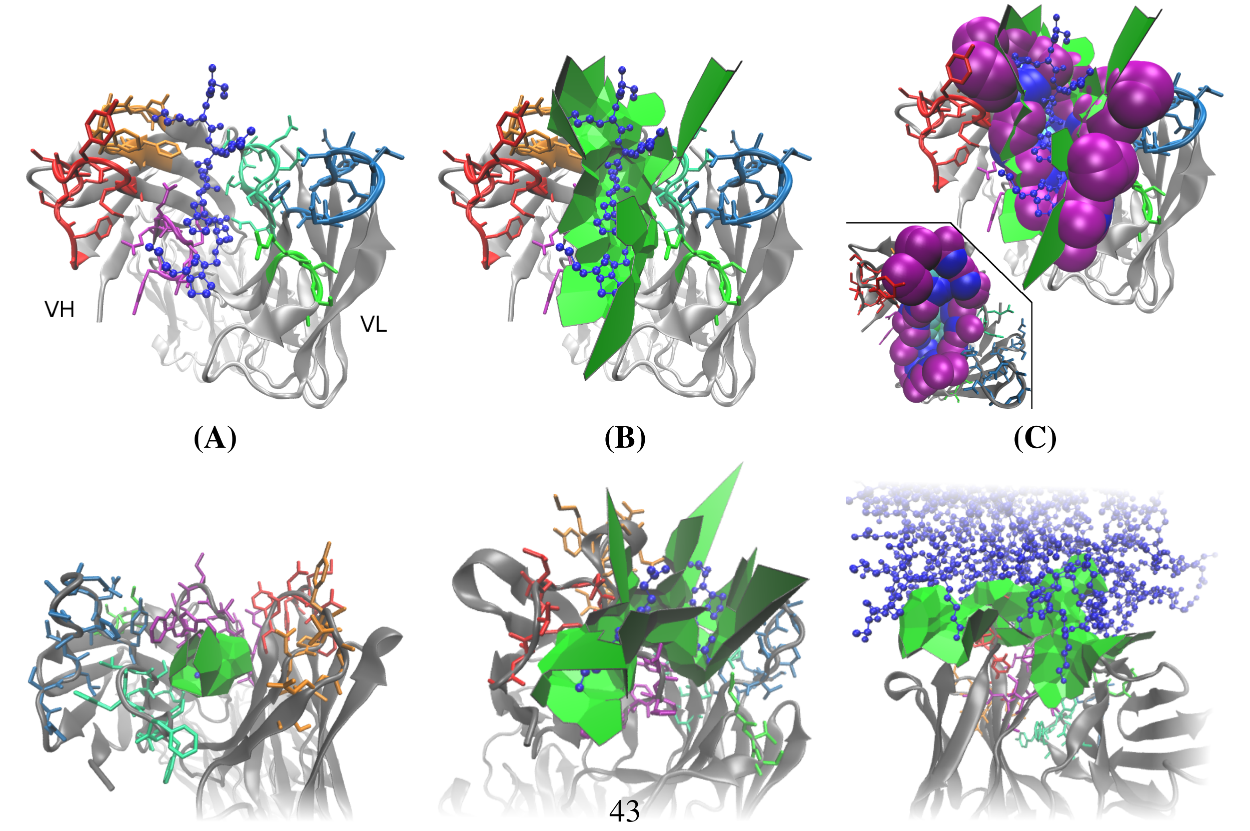

(D: 2O5X) **(E: 2HKF)** **(F: 4OGY)**

A.4 Characteristics of the binding patch predict the ligand type

Solvent accessibility properties of binding patches exhibit a broken symmetry between the Ig and Ag side. In Fig. S4, there is a strong correlation between BSA and $|\mathscr{I}|$, a very-well known fact. However,this is less obvious when considering only Ig atoms or Ag atoms. This shows how the shape complementarity between binding patches results in an overall balance between the BSA and the number of atoms at interface.

Fig. S 4 Buried Surface Area versus number of interface atoms: whole interface, Ig side, Ag side. The well-known strong correlation between BSA() and $|\mathscr{I}|$ (panel (a)) gets weaker when considering the Ig (panel (b)) and the Ag sides (panel (c)) separately. The Pearson coefficients obtained are equal to 0.99, 0.82 and 0.89 in cases (a,b,c).

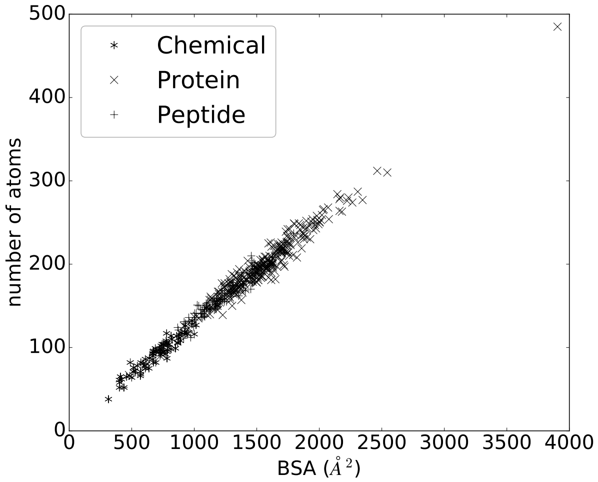

(a) BSA versus $|\mathscr{I}|$.

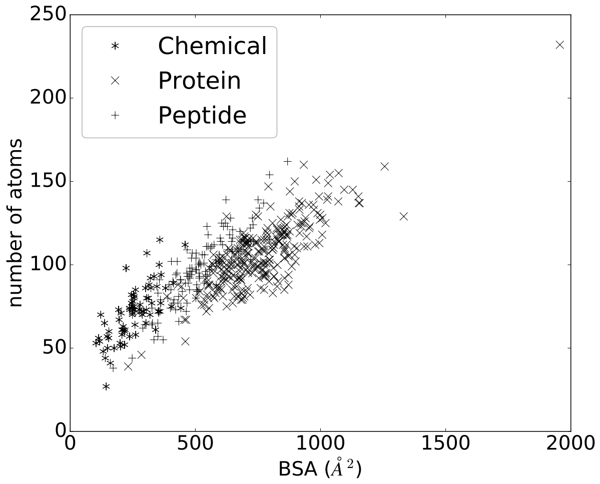

(b) $\mathbf{BSA_{Ig}}$ versus $|\mathscr{I}_{\mathbf{Ig}}|$.

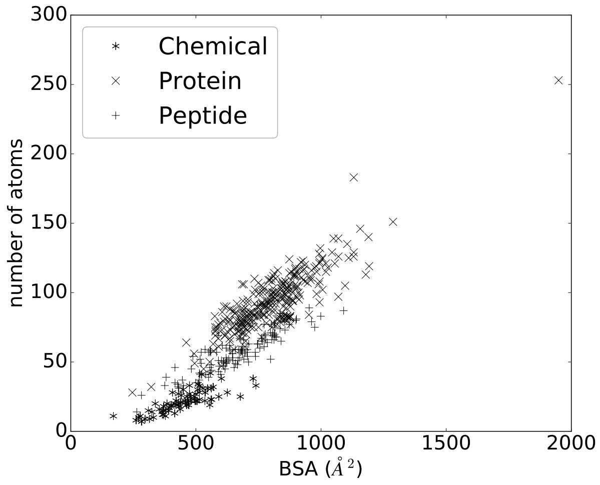

(c) $\mathbf{BSA_{Ag}}$ versus $|\mathscr{I}_{\mathbf{Ag}}|$.

Solvent accessibility asymmetry is a signature for the ligand type. We feed two descriptors ($\overline{\text{bsa}}_{\text{Ag}}$ and $\overline{\text{bsa}}_{\text{Ig}}$) to a classifier in order predict the ligand type of a complex. These descriptors are computed using the `sbl-intervor-ABW-atomic.exe` binary from the structural bioinformatics

library (SBL, sbl.inria.fr). The classifier is a decision tree from the R package rpart.

We first compute the in-sample classification error. This error is obtained by classifying the training set and results in optimistic error rates. We therefore compute a *cross-validated* error rate. To this end, we randomly divide the dataset in five subsets of equal size and alternatively use four subsets to classify the fifth. At the end of this procedure, each sample has been predicted and the proportion of misclassified samples can be computed. Since the data is partitioned randomly, we repeat the above procedure 1000 times and compute the average confusion matrix(Table SIV) and both the overall and per class error rates.

The resulting median and average error rates per ligand type are the following: chemical: 5%, 6%; peptide: 19%, 19% ; protein: 7%, 7%. The overall median and average error rates are 9.6% and 9.7% respectively.

Since the data is not balanced, *i.e.* some ligand types are over-represented compared to others we check whether keeping a balanced proportion of classes in each fold would yield differing results. The resulting median and average error rates per ligand type are the following: chemical: 5%, 6%; peptide: 20%, 20% ; protein: 7%, 6%; and the overall median and average error rates are 9.6% and 9.7% respectively, which is essentially similar to the non-balanced cross-validation.

The classification rules resulting from the decision tree run on the whole dataset (i.e. no-cross-validation) are the following (Fig. S5) : $\overline{\mathrm{bsa}}_{\mathrm{Ag}} \geq 14.3 \Rightarrow$ chemical ligand; $10.7 \leq \overline{\mathrm{bsa}}_{\mathrm{Ag}} < 14.3 \Rightarrow$ peptide ligand; $\overline{\mathrm{bsa}}_{\mathrm{Ag}} < 10.7$ AND $\overline{\mathrm{bsa}}_{\mathrm{Ig}} < 5.75 \Rightarrow$ peptide ligand; $\overline{\mathrm{bsa}}_{\mathrm{Ag}} < 10.7$ AND $\overline{\mathrm{bsa}}_{\mathrm{Ig}} \geq 5.75$ $\Rightarrow$ protein ligand.

Fig. S 5 Classification rules characterizing the binding patch depending on the ligand types. See supplemental text for details. The classification rules are: $\overline{\text{bsa}}_{\text{Ag}} \geq 14.3 \Rightarrow$ chemical ligand; $10.7 \leq \overline{\text{bsa}}_{\text{Ag}} < 14.3 \Rightarrow$ peptide ligand; $\overline{\text{bsa}}_{\text{Ag}} < 10.7$ AND $\overline{\text{bsa}}_{\text{Ig}} < 5.75 \Rightarrow$ peptide ligand; $\overline{\text{bsa}}_{\text{Ag}} < 10.7$ AND $\overline{\text{bsa}}_{\text{Ig}} \geq 5.75 \Rightarrow$ protein ligand. The three lines of a box read as follows: tow row: majority ligand type (chemical, peptide, protein); middle row: fraction for the three classes; bottom row: percentage of the whole dataset.

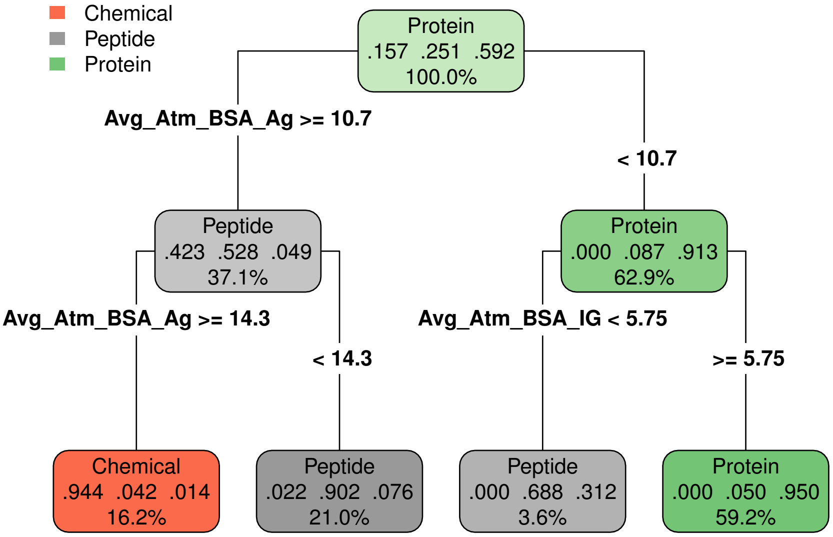

Table S IV Average confusion matrix for ligand type prediction. Results obtained by running 5-fold cross-validation 1000 times. Each repetition results in a confusion matrix which is averaged– e.g. on average 4.6 chemicals out of 77 are predicted as peptides.

Predicted \ Actual	Chemical	Peptide	Protein
Chemical	72.4	3.0	1.0
Peptide	4.6	99.1	17.9
Protein	0.0	20.9	270.1

Table S V Median BSA and median of BSA/BSA_{Ig} per species and per ligand type. Median BSA contributed to the interface by different parts of the Ig, for various ligand types and species. Percentages relative to the BSA of the whole Ig are included in parentheses.

	Human			Mouse			Other		
	Chemical	Peptide	Protein	Chemical	Peptide	Protein	Chemical	Peptide	Protein
Ig	143.7 (100.0%)	645.7 (100.0%)	812.0 (100.0%)	254.6 (100.0%)	486.6 (100.0%)	703.9 (100.0%)	275.0 (100.0%)	546.6 (100.0%)	767.4 (100.0%)
VH	89.8 (57.0%)	418.3 (66.2%)	569.9 (67.7%)	154.8 (59.9%)	287.0 (58.4%)	471.1 (64.3%)	141.1 (54.4%)	307.5 (54.0%)	462.8 (63.7%)
VH CDR	49.4 (39.6%)	381.3 (56.9%)	510.7 (62.1%)	128.3 (46.6%)	236.8 (49.5%)	434.9 (57.4%)	116.2 (42.3%)	241.3 (50.1%)	420.0 (58.9%)
VH CDR1	10.3 (7.5%)	63.0 (11.1%)	86.2 (9.5%)	19.0 (8.5%)	46.5 (10.0%)	101.7 (14.7%)	23.6 (7.7%)	37.6 (8.2%)	54.5 (8.0%)
VH CDR2	0.0 (0.0%)	76.3 (12.6%)	139.0 (17.4%)	13.0 (5.0%)	61.4 (13.2%)	99.8 (14.4%)	0.0 (0.0%)	55.9 (13.8%)	148.9 (20.5%)
VH CDR3	44.0 (26.9%)	180.3 (29.3%)	233.9 (30.4%)	76.9 (29.4%)	117.4 (24.1%)	208.7 (29.1%)	97.3 (35.4%)	108.5 (20.7%)	193.2 (26.1%)
VH FR	25.8 (11.7%)	24.5 (4.7%)	30.6 (3.4%)	24.0 (7.8%)	42.6 (8.4%)	28.8 (4.3%)	15.0 (6.9%)	38.2 (7.1%)	48.7 (7.6%)
VH FR1	0.0 (0.0%)	0.0 (0.0%)	0.0 (0.0%)	0.0 (0.0%)	0.0 (0.0%)	0.0 (0.0%)	0.0 (0.0%)	0.0 (0.0%)	0.0 (0.0%)
VH FR2	8.1 (5.3%)	6.8 (1.6%)	0.0 (0.0%)	11.9 (3.8%)	22.5 (4.4%)	6.6 (0.9%)	11.3 (3.7%)	15.5 (2.9%)	0.1 (0.0%)
VH FR3	0.0 (0.0%)	15.5 (2.1%)	18.1 (2.5%)	0.0 (0.0%)	10.8 (2.3%)	15.7 (2.4%)	0.0 (0.0%)	11.5 (2.1%)	29.2 (3.5%)
VH FR4	0.1 (0.1%)	0.0 (0.0%)	0.0 (0.0%)	0.0 (0.0%)	0.0 (0.0%)	0.0 (0.0%)	0.0 (0.0%)	0.0 (0.0%)	0.0 (0.0%)
VH OTHER	0.0 (0.0%)	0.0 (0.0%)	0.0 (0.0%)	0.0 (0.0%)	0.0 (0.0%)	0.0 (0.0%)	0.0 (0.0%)	0.0 (0.0%)	0.0 (0.0%)
VL	71.2 (43.0%)	185.6 (33.8%)	261.6 (32.3%)	104.2 (40.1%)	190.6 (41.6%)	276.3 (35.7%)	132.8 (45.6%)	203.7 (46.0%)	292.9 (36.3%)
VL CDR	46.3 (30.2%)	162.9 (33.4%)	211.1 (26.9%)	92.5 (37.4%)	175.3 (38.2%)	224.5 (30.4%)	113.6 (41.3%)	196.4 (37.5%)	247.2 (32.6%)
VL CDR1	0.0 (0.0%)	46.5 (9.2%)	74.7 (8.3%)	32.4 (13.3%)	76.6 (15.7%)	52.5 (7.9%)	13.5 (4.9%)	49.5 (11.7%)	86.1 (12.8%)
VL CDR2	0.0 (0.0%)	0.0 (0.0%)	3.9 (0.5%)	0.0 (0.0%)	0.0 (0.0%)	10.0 (1.5%)	0.0 (0.0%)	0.0 (0.0%)	8.7 (0.9%)
VL CDR3	45.0 (23.7%)	114.9 (17.9%)	98.1 (12.1%)	52.9 (22.4%)	101.7 (20.6%)	103.3 (16.0%)	94.4 (34.2%)	124.6 (23.2%)	111.4 (14.5%)
VL FR	25.0 (16.3%)	0.0 (0.0%)	11.1 (1.3%)	0.0 (0.0%)	0.0 (0.0%)	6.8 (0.9%)	3.7 (1.2%)	16.8 (2.8%)	2.8 (0.4%)
VL FR1	0.0 (0.0%)	0.0 (0.0%)	0.0 (0.0%)	0.0 (0.0%)	0.0 (0.0%)	0.0 (0.0%)	0.0 (0.0%)	0.0 (0.0%)	0.0 (0.0%)
VL FR2	15.4 (9.5%)	0.0 (0.0%)	0.0 (0.0%)	0.0 (0.0%)	0.0 (0.0%)	0.0 (0.0%)	3.7 (1.2%)	16.2 (2.7%)	0.0 (0.0%)
VL FR3	0.0 (0.0%)	0.0 (0.0%)	0.9 (0.1%)	0.0 (0.0%)	0.0 (0.0%)	0.0 (0.0%)	0.0 (0.0%)	0.0 (0.0%)	0.0 (0.0%)
VL FR4	0.0 (0.0%)	0.0 (0.0%)	0.0 (0.0%)	0.0 (0.0%)	0.0 (0.0%)	0.0 (0.0%)	0.0 (0.0%)	0.0 (0.0%)	0.0 (0.0%)
VL OTHER	0.0 (0.0%)	0.0 (0.0%)	0.0 (0.0%)	0.0 (0.0%)	0.0 (0.0%)	0.0 (0.0%)	0.0 (0.0%)	0.0 (0.0%)	0.0 (0.0%)

Fig. S 6 Human and mouse VH CDR length versus BSA. The [CDR1. CDR2] length are characteristic of the different *Homo sapiens* and *Mus musculus* VH subgroups. There are highly varying levels of BSA for CDR of the same length. The information given by the length of a CDR is therefore not sufficient to infer its contribution to the interface.

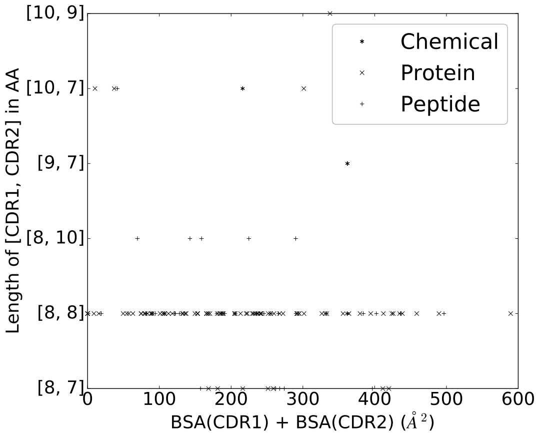

(a) Human [VH CDR1. VH CDR2]. Five complexes are discarded because of aberrant VH CDR1 and VH CDR2 lengths

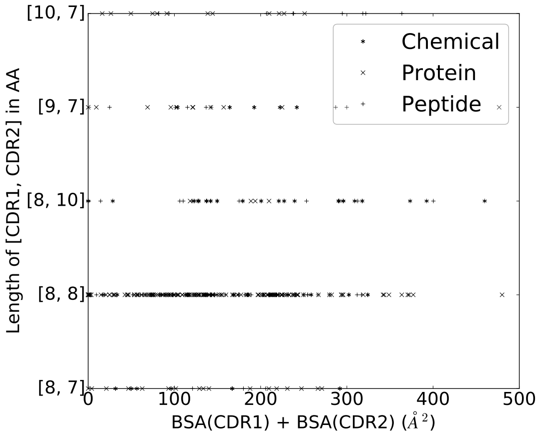

(b) Mouse [VH CDR1. VH CDR2].

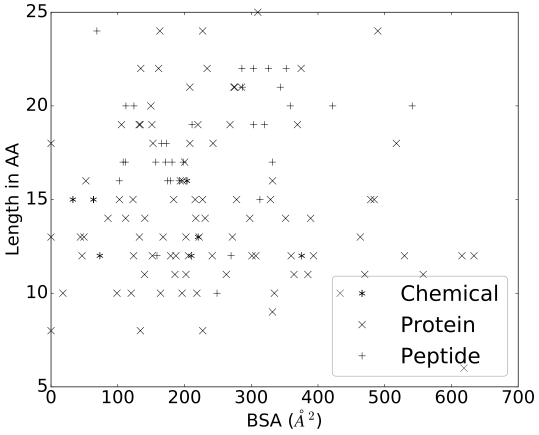

(c) Human VH CDR3. Twelve complexes are discarded because of aberrant VL CDR1 and VL CDR2 lengths

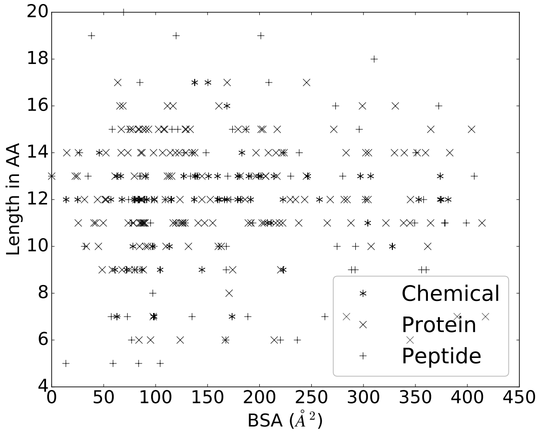

(d) Mouse VH CDR3.

Fig. S 7 Human and mouse VL CDR length versus BSA. The human [CDR1.CDR2] lengths [6.3] characterize both V-kappa and V-lambda. The other lengths characterize either V-kappa ([7.3], [11.3] and [12.3]) or V-lambda ([8.3] and [9.3]). The mouse [CDR1.CDR2] lengths [7.7] and [9.3] characterize V-lambda. The other lengths characterize V-kappa. There are highly varying levels of BSA for CDR of the same length. The information given by the length of a CDR is therefore not sufficient to infer its contribution to the interface.

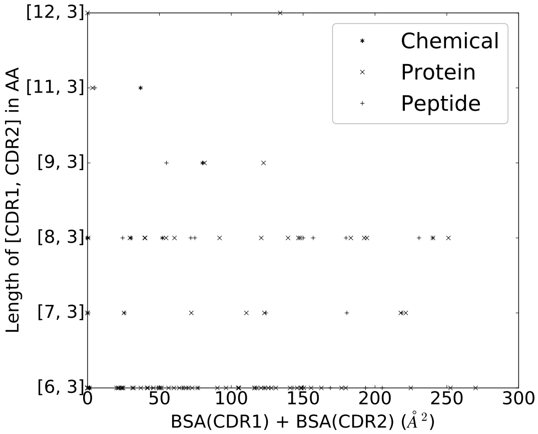

(a) Human [VL CDR1. VL CDR2].

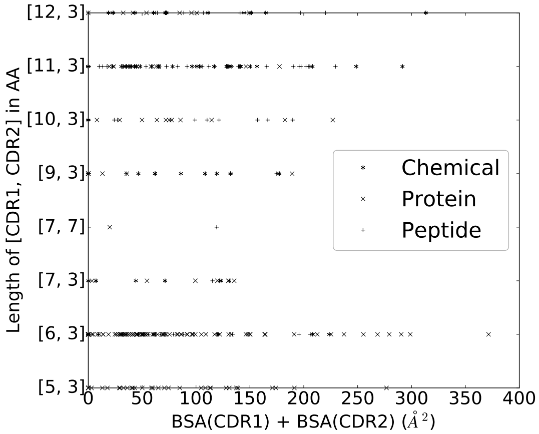

(b) Mouse VL CDR1 and VL CDR2.

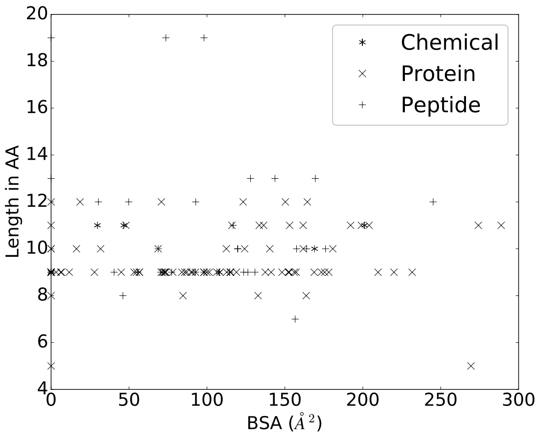

(c) VL CDR3, Human.

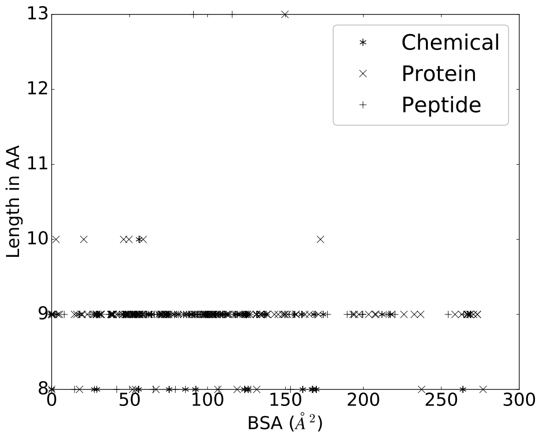

(d) VL CDR3, Mouse.

Complexes with free VH CDR1 or VH CDR2 are not uncommon since they occur for 45/489 (∼9%) and 50/489 (∼10%) complexes, respectively (5b). In contrast, this is rare for VH CDR3 (6/489 occurrences, ∼1%).

On the other hand, 242/489 (∼49%) complexes involve a free VL CDR2 (5c), a fact to be

interpreted in the context of a lesser length variability and, as we shall see, a location on the side of the domain. 70/489 ($\sim$14%) complexes have an free VL CDR1 and 34/489 ($\sim$7%) complexes have an free VL CDR3 which is more than five time as much as VH CDR3.

Fig. S 8 a) and b): IVW-IPL of the CDR of VH and VL respectively. c): Variation of the atomic volume as a function of the shelling order. Atoms with a higher shelling order tend to be more packed. The rise after shelling order 4 is likely due to a much smaller number of atoms since 1) interfaces with deeply buried atoms are rare, 2) only a limited number of atoms can be deeply buried in an interface.

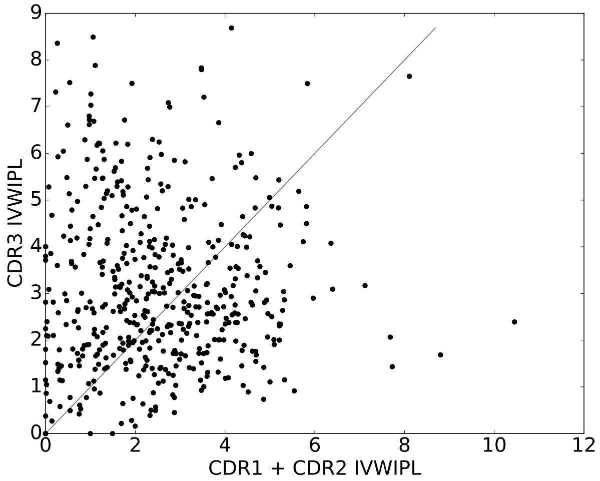

(a)

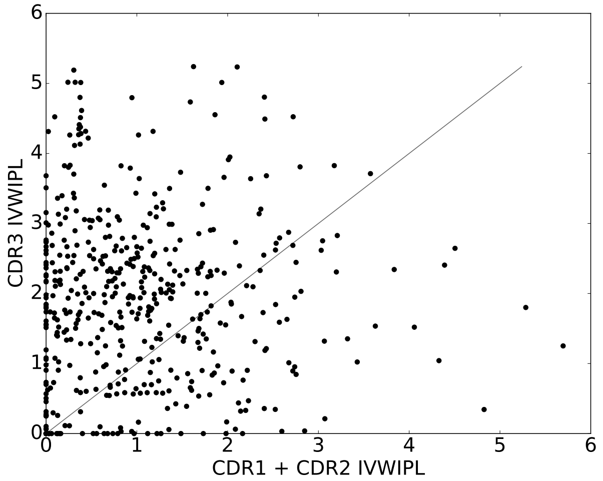

(b)

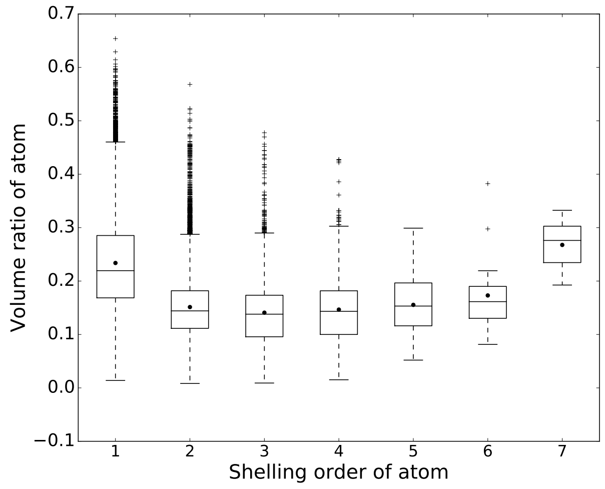

(c)

A.5 Binding affinity predictions

Ig - Ag complexes. The structure affinity benchmark (SAB) [6] contains 17 Ig - Ag cases (PDB IDs: 1AHW, 1BJ1, 1BVK, 1DQJ, 1E6J, 1FSK, 1IQD, 1JPS, 1MLC, 1NCA, 1NSN, 1P2C, 1VFB, 1WEJ, 2JEL, 2VIR and 2VIS). However, 1IQD and 1NSN are discarded as only an upper bound on their K_d is provided in the SAB. Furthermore, 1E6J is also discarded because too many atoms could not be matched between the bound and unbound structures. Finally 1ZLI is removed from the training set because too many atoms could not be matched between the bound and unbound structures and 1UUG is also removed from the training set because of only an upper bound on its K_d is provided.

Note that this leaves 14 Ig - Ag cases to predict, using a learning set involving $144-2-3-14=125$ complexes.

Affinity estimation as a regression problem. In this work, we estimate binding affinities using k nearest neighbors regression (knn) [7, 8], a non parametric strategy which does not require any a priori on the mathematical model for the response variable estimated – as opposed to say linear regression.

To describe knn regression, which is a two step strategy, recall that we model complexes using two parameters denoted IVW-IPL and $\text{NIS}^{charged}$ (see main text); these variables define a parameter space denoted $\mathscr{C}$. As a pre-processing step, we compute the parameters IVW-IPL and $\text{NIS}^{charged}$ for the training set (125 cases), yielding a point cloud P in $\mathscr{C}$ (Fig. 3). To estimate the affinity a complex q, we proceed in two steps. First, the k nearest neighbors of q in P are sought, with k a predefined number. Second, the affinity of q is estimated by averaging those of its k nearest neighbors.

We assess the quality of our predictions in two ways:

- First, by varying the value k. From a theoretical standpoint [7], it is known that k must be

super-logarithmic and sub-linear in the number of cases processed. Since $log(144) \sim 5$, we explore the range $k \in 5,\ldots,25$ (Fig. 4). Practically, the results discussed in the main text correspond to $k = 10$.

- Second, by comparing the estimates against those yielded by the linear model using the same variables. This model corresponds to the one defined in [9]. See Table SVI for this comparison.

Practically, we compute the variables used by the regression method using the `binding affinity prediction` package from the structural bioinformatics library (SBL, `sbl.inria.fr`). For the fitting, we use the scikit-learn library [10], namely the `neighbors` package for knn regression, and the `linear_model` package for the least-squares linear regression.

Results. The median absolute error does not vary much between 8 and 12 neighbors (from 1.008 to 0.898, Fig. 4). Moreover, the error for 10 neighbors is close to the median error for the total range considered (0.878 compared to 0.910 kcal/mol).

Statistics for the KNN predictions: Pearson's correlation coefficient: 0.488 (p-value = 0.077). Median absolute error: 0.878. Number of complexes with errors below 1, 2, and 3 orders of magnitude respectively: 8, 13, 3.

Statistics for predictions: obtained with the linear model from [9]: Pearson's correlation coefficient: 0.326 (p-value = 0.255). Median absolute error: 1.017. Number of complexes with errors below 1, 2, and 3 orders of magnitude respectively: 9, 13, 14.

Table S VI Binding affinities: experimental values against those obtained with two regression methods. KNN predictions: obtained using the *k*-nearest neighbors regressor, as explained in the text; linear model predictions: obtained with the linear model introduced in [9].

PDB ID	Experimental -ΔG	KNN predictions	linear model predictions
1AHW	11.55	11.938	11.296
1BJ1	11.55	12.543	12.093
1BVK	10.53	7.902	10.699
1DQJ	11.67	11.839	12.522
1FSK	13.12	12.515	11.526
1JPS	13.64	12.159	11.943
1MLC	9.61	10.374	11.293
1NCA	11.02	12.61	11.326
1P2C	13.63	11.728	11.882
1VFB	11.46	11.792	12.354
1WEJ	12.48	11.891	11.338
2JEL	11.59	12.142	11.580
2VIR	12.28	7.902	11.120
2VIS	7.36	8.794	11.306

Fig. S 9 Binding affinity analysis and predictions for Ig - Ag complexes. Predictions performed by the linear model from [9]. Dashed, dash-dotted and dotted lines respectively show errors of ±1.4, ±2.8, ±4.2 kcal/mol, corresponding to K_d approximated within one, two and three orders of magnitude.

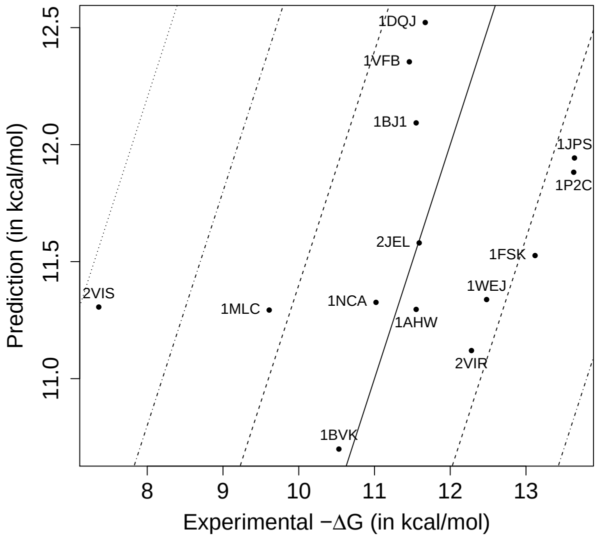

Fig. S 10 Comparison between this work and the PRODIGY server. The vertical dashed lines materialize the experimental values of the complexes. Labels are positioned next to the corresponding red dot.

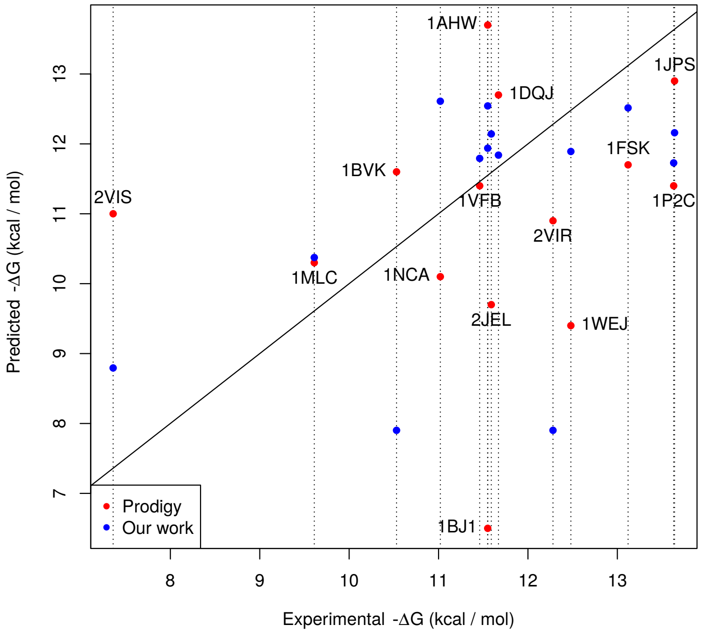

Fig. S 11 Prediction error versus average distance of the 10 nearest-neighbors and the standard deviation of their affinity values.

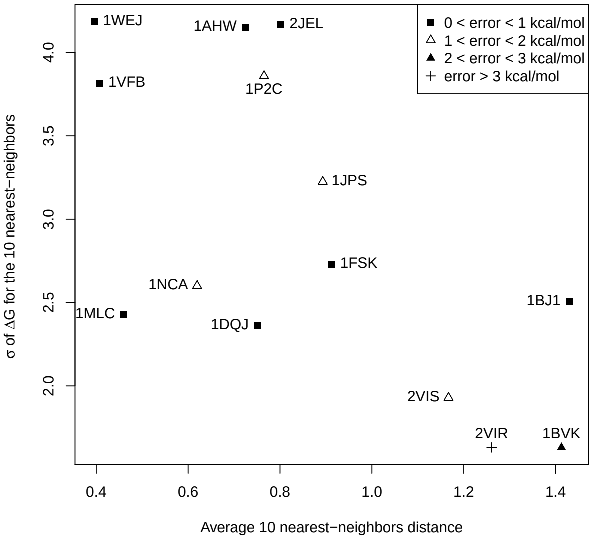

References

[1] F. Ehrenmann, Q. Kaas, and M-P. Lefranc. IMGT/3Dstructure-DB and IMGT/DomainGapAlign: a database and a tool for immunoglobulins or antibodies, T cell receptors, MHC, IgSF and MhcSF. *Nucl. Acids Res.*, 38:D301–307, 2010.

[2] M-P. Lefranc, C. Pommié, M. Ruiz, V. Giudicelli, E. Foulquier, L. Truong, V. Thouvenin-Contet, and G. Lefranc. IMGT unique numbering for immunoglobulin and T cell receptor variable domains and Ig superfamily V-like domains. *Developmental & Comparative Immunology*, 27(1):55–77, 2003.

[3] M-P. Lefranc and G. Lefranc. *The immunoglobulin FactsBook*. Academic Press, 2001.

[4] F. Cazals. Revisiting the Voronoi description of protein-protein interfaces: Algorithms. In T. Dijkstra, E. Tsivtsivadze, E. Marchiori, and T. Heskes, editors, *International Conference on Pattern Recognition in Bioinformatics*, pages 419–430, Nijmegen, the Netherlands, 2010. Lecture Notes in Bioinformatics 6282.

[5] F. Cazals, F. Proust, R. Bahadur, and J. Janin. Revisiting the Voronoi description of protein-protein interfaces. *Protein Science*, 15(9):2082–2092, 2006.

[6] P.L. Kastritis, I.H. Moal, H. Hwang, Z. Weng, P.A. Bates, A. Bonvin, and J. Janin. A structure-based benchmark for protein-protein binding affinity. *Protein Science*, 20:482–491, 2011.

[7] L. Györfi and A. Krzyzak. *A distribution-free theory of nonparametric regression*. Springer, 2002.

[8] G. Biau and L. Devroye. *Lectures on the nearest neighbor method*. Springer, 2015.

[9] S. Marillet, P. Boudinot, and F. Cazals. High resolution crystal structures leverage protein binding affinity predictions. *Proteins: structure, function, and bioinformatics*, 1(84):9–20, 2015.

[10] F. Pedregosa, G. Varoquaux, A. Gramfort, V. Michel, B. Thirion, O. Grisel, M. Blondel, P. Prettenhofer, R. Weiss, V. Dubourg, J. Vanderplas, A. Passos, D. Cournapeau, M. Brucher, M. Perrot, and E. Duchesnay. Scikit-learn: Machine learning in Python. *Journal of Machine Learning Research*, 12:2825–2830, 2011.
